# A systems biology approach to unveil shared therapeutic targets and pathological pathways across major human cancers

**DOI:** 10.1016/j.csbj.2025.11.061

**Published:** 2025-11-29

**Authors:** Aftab Alam, Mohd Faizan Siddiqui, Rifat Hamoudi, Uday Kishore, Maria J. Fernandez-Cabezudo, Basel K. Al-Ramadi

**Affiliations:** a Department of Medical Microbiology and Immunology, College of Medicine and Health Sciences, United Arab Emirates University, Al Ain, United Arab Emirates; b International Medical Faculty, Osh State University, Osh City, Kyrgyz Republic, Kyrgyzstan; c Research Institute for Medical and Health Sciences, University of Sharjah, Sharjah P.O. Box 27272, United Arab Emirates; d Department of Clinical Sciences, College of Medicine, University of Sharjah, Sharjah P.O. Box 27272, United Arab Emirates; e Division of Surgery and Interventional Science, University College London, London, United Kingdom; f Department of Veterinary Medicine (CAVM), United Arab Emirates University, Al Ain, United Arab Emirates; g Zayed Center for Health Sciences, United Arab Emirates University, Al Ain, United Arab Emirates; h Department of Biochemistry and Molecular Biology, College of Medicine and Health Sciences, United Arab Emirates University, Al Ain, United Arab Emirates; i ASPIRE Precision Medicine Research Institute, Abu Dhabi, United Arab Emirates University, Al Ain, United Arab Emirates

**Keywords:** TCGA, Pan-cancer, Multi-omics, WGCNA, PPI network, Hub genes, Prognostic biomarkers, Prognostic analysis, Risk score model, Machine learning (ML)

## Abstract

Cancer remains a major cause of global mortality. Despite their distinct clinical and molecular characteristics, different cancer types often share fundamental molecular mechanisms that remain underexplored. In this study, we systematically profiled transcriptomic data from four highly prevalent cancers, including breast, lung, colorectal, and prostate, to uncover shared molecular signatures with diagnostic and prognostic value. Using The Cancer Genome Atlas (TCGA) datasets and a rigorous integrative workflow, we combined differential expression analysis, Elastic Net–based feature selection, and weighted gene co-expression network analysis (WGCNA) to identify 179 cross-cancer signature genes linked to clinical traits. Protein–protein interaction (PPI) analysis and Markov Cluster Algorithm (MCL) clustering further refined these into 26 robust hub genes with strong diagnostic potential. Survival analyses demonstrated that these hub genes possess strong prognostic potential, while pan-cancer assessment revealed consistent dysregulation across more than 20 cancer types. Several hub genes also displayed context-dependent immunomodulatory roles within the tumor microenvironment. Notably, 16 hub genes showed strong associations with metastatic disease: some were consistently downregulated, suggesting tumor-suppressive functions, whereas others were upregulated in a cancer-specific manner, reflecting context-dependent oncogenic roles. We constructed a hub gene-based signature and demonstrated its potential as a prognostic marker in these four cancers types. This comprehensive analysis offers valuable insights into shared oncogenic mechanisms, contributing to improved diagnosis, prognosis, and targeted therapies across multiple cancer types.

## Graphical Abstract

**Figure:**
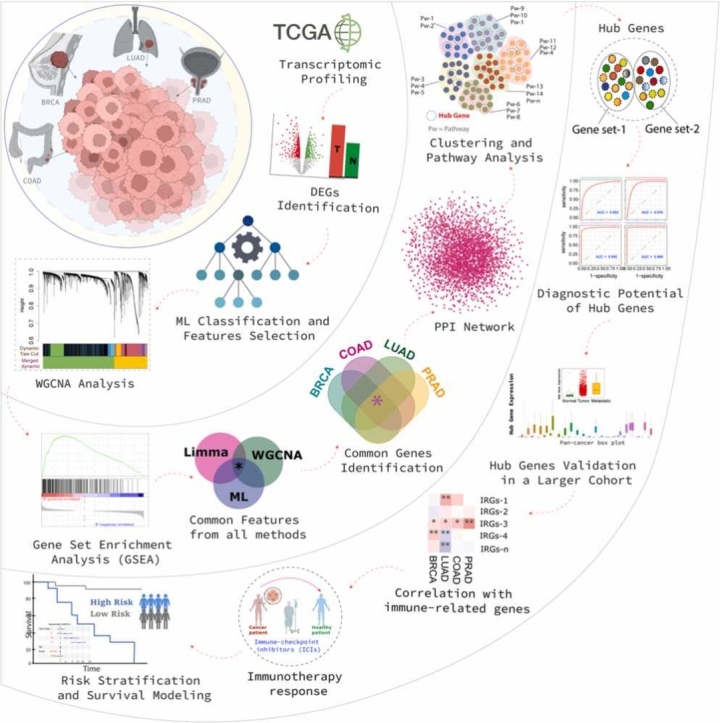
Schematic overview of the study workflow, illustrating the key steps of data collection, preprocessing, analysis, and validation.

## 1 Introduction

Cancer remains a major global public health challenge, driven by aging populations, lifestyle factors, infections, and environmental exposures. It excessively affects low- and middle-income countries (LMICs), which face limited access to prevention, diagnosis, and treatment. In 2022, an estimated 20 million new cases and 9.7 million cancer deaths occurred globally, with nearly 70 % of deaths in LMICs. By 2050, cancer cases are projected to rise by 87.5 %, placing even more pressure on health systems, individuals, and communities [1]. According to the World Health Organization (WHO), the most common cancers are breast, lung, colon, and prostate carcinomas (hereafter termed selected cancer types, SCTs), which were chosen for this study not only because they are highly prevalent but also because they all arise from epithelial tissues [2], [3]. This shared epithelial origin provides a biological basis for comparing them, as it underlies common molecular and phenotypic features despite differences in tissue of origin. By studying these cancers together, we aim to identify shared molecular signatures that can improve early diagnosis and guide therapeutic strategies, ultimately providing a framework for understanding potential common biomarkers and pathways in epithelial-derived cancer.

Global cancer statistics highlight that breast cancer is the most common cancer among women, making up 12.5 % of all new cancer cases globally. In 2022, 2.3 million women were diagnosed with breast cancer, resulting in 670,000 deaths [4]. Lung cancer affects ∼2.5 million people each year and claims 1.8 million lives annually [5]. Colorectal cancer, with > 1.9 million diagnoses in 2022, is the third most common cancer globally, responsible for more than 900,000 deaths each year. Prostate cancer affects 1 in 8 men during their lifetime and resulted in 1.4 million diagnoses and 375,000 deaths in 2020 [6]. To reduce the cancer burden, early detection through screening and prompt treatment improves survival rates. Biomarker detection plays a critical role in this process by identifying cancer at an early stage, confirming its type, and guiding treatment choices. Specific biomarkers can also help predict the aggressiveness of the tumor and its response to treatments, enabling personalized therapy.

High-throughput gene expression technologies offer extensive genetic insights into cancer types, revealing crucial changes in disease progression [7], [8], [9], [10]. By leveraging genomics, epigenomics, and transcriptomics data available from online databases, it is possible to uncover potential molecular signatures associated with cancer [11], [12], [13], [14]. Advances in precision medicine, combined with the TCGA/GEO/Other database, enable researchers to link genomic features with clinical outcomes and identify new diagnostic and therapeutic targets. For example, Liu et al. investigated stomach adenocarcinoma (STAD) to identify molecular biomarkers for prognosis and targeted therapy using TCGA data [15]. In another study, researchers studied colon cancer to identify immune-related lncRNAs (IRLs) as prognostic biomarkers using Gene Expression Omnibus (GEO) and TCGA datasets [16]. A similar study by *Gao et al.* identified potential early diagnostic and prognostic biomarkers for liver cancer by analyzing TCGA RNA-seq and clinical data [17].

Recently, machine learning (ML) models, including support vector machines (SVMs), random forests, linear models, and Elastic Net models (ENM), have emerged as effective tools for predicting gene signatures [18], [19], [20]. Convolutional neural networks (CNNs), widely used in cancer diagnostics, have recently been applied in an innovative way to transform tissue images into cell-based graphs, representing each cell as a node connected to its neighbors for detailed tissue analysis [21]. Interpretable ML (IML) approaches, such as SHapley Additive exPlanations (SHAP), have also been employed to predict the risk of cardiovascular disease and cancer from dietary antioxidants [22]. Progress demonstrating how ML can support disease prediction, biomarker discovery, and treatment optimization was recently reviewed [23], highlighting its potential to advance precision medicine

Collectively, these approaches hold promise for improving our understanding, diagnosis, and management of cancer. Nevertheless, despite significant advances in high-throughput technologies and data integration approaches, identifying common molecular signatures remains challenging due to the inherent heterogeneity of cancer. Most existing studies focus on individual cancer types, which limits our understanding of shared oncogenic processes that could be leveraged for pan-cancer strategies. This creates a critical research gap in systematically identifying and functionally characterizing robust, context-dependent molecular signatures that transcend individual cancer boundaries, and provide clinically relevant potential biomarkers and therapeutic targets across prevalent cancers.

Pan-cancer studies have revealed common molecular features across cancers, but they often use linear analysis methods. For example, some studies identify candidate genes through differential expression across multiple cancers [24], or concentrate on individual cancer types [25], [26], [27], [28], while others deeply analyze pre-selected genes [29], [30], [31]. Although these methods are useful, but can be biased either by arbitrary thresholds in gene selection or by focusing only on known genes.

We present a cross-cancer, multi-level co-identification framework designed to more systematically analyze shared gene sets and help reveal potential universal oncogenic drivers. We systematically analyzed four major malignancies (e.g., SCTs) to uncover conserved DEGs shared across them. The central innovation of our study lies in a multi-level strategy for identifying and validating these shared DEGs. First, we applied a consensus approach that retained only those DEGs detected by both univariate (limma) and multivariate (Elastic Net) analyses within the SCTs, ensuring robustness. These common DEGs were then validated using independent transcriptomic datasets, confirming their reproducibility and strong prognostic potential.

Using WGCNA [32], on the pre-validated cross-cancer DEG set, we identified key genes within co-expression modules that consistently act as central players in these SCTs. Following the common co-identification of DEGs through limma, Elastic Net, and WGCNA, we examined their topological roles within the global interactome using PPI network analysis, revealing functionally coherent clusters dominated by a few hub genes. These hub genes, characterized by high network centrality, were further validated across independent transcriptomic and proteomic datasets, as well as TCGA pan-cancer data, confirming both their reproducibility and prognostic significance. We also assessed the prognostic relevance of these hub genes for overall survival and risk stratification. Finally, we investigated their impact on the tumor immune microenvironment, demonstrating that these hub genes may consistently influence immune responses in SCTs.

Overall, this study presents a robust, integrative framework for identifying a conserved hub gene set that serves as a universal driver of tumorigenesis, with important implications for the diagnosis and treatment of SCTs. Nonetheless, experimental validation is required to confirm these findings and assess their potential clinical applications.

## 2 Materials and methods

### 2.1 Data collection and differential expression analysis

We performed transcriptomic profiling analysis on four cancer types, including breast invasive carcinoma (BRCA), lung adenocarcinoma (LUAD), colon adenocarcinoma (COAD), and prostate adenocarcinoma (PRAD). The RNA-Seq data for these cancer types were obtained using the **TCGAbiolinks** R package (v. 2.37.1) [33]. The differential expression analysis (DEA) between ‘*Primary Tumor’* and ‘*Solid Tissue Normal’* samples was performed using the limma package, a commonly used tool for RNA-Seq data analysis. Raw gene count data were normalized using the TMM (Trimmed Mean of M-values) method to account for differences in library sizes and sequencing depth across samples. The normalization process was carried out using the calcNormFactors() function, followed by the voom transformation, which converted the count data into log-counts per million (log-CPM) and estimated the mean-variance relationship for each gene. Next, a design matrix was built using the *model.matrix()* function based on the condition variable "definition" (e.g., tumor vs. normal).

The analysis included 1111 tumor and 113 normal samples for BRCA, 539 tumor and 59 normal samples for LUAD, 481 tumor and 41 normal samples for COAD, and 501 tumor and 52 normal samples for PRAD. The DEA was performed using *lmFit()* and *eBayes()* from the limma package (v. 3.58.1). DEGs were identified using a threshold of absolute fold change (|FC=) > 1.5 and an adjusted *P* < 0.05. The fold change threshold was applied to ensure selected genes had a biologically meaningful effect size, while the Benjamini-Hochberg (BH) adjusted *P* controlled the false discovery rate (FDR) in multiple hypothesis testing. To define a shared transcriptional signature, we analyzed primary solid tumors and corresponding normal tissues across multiple SCTs. Unsupervised PCA revealed a clear separation between tumor and normal samples, indicating distinct transcriptomic profiles (Suppl. Fig. 1** A**). Differential expression analysis identified a comprehensive set of DEGs, visualized by volcano plots (Suppl. Fig. 1B). This list was further refined to a core set of common DEGs consistently dysregulated across SCTs, as demonstrated by their expression patterns and overlap in a venn diagram (Suppl. Fig. 2A**–B**). The relevance of this common gene set was subsequently validated in an independent GEO cohort (Suppl. Fig. 2** C**).

### 2.2 Machine learning classification

We explored a machine learning approach to classify unseen samples as either tumor or non-tumor, starting with a simple linear model with feature selection, followed by an Elastic Net model [34], which is a regularized logistic regression that performs automatic feature selection while classifying samples as tumor or non-tumor. Here, we used expression data that has already been normalized, along with clinical features such as tumor vs. normal status were included for each SCT. For each SCT, we split the samples into a training set (75 %) and a test set (25 %) using stratified sampling with the *createDataPartition()* function from the caret package (v. 7.0.1) to preserve class distribution balance. We then trained a regularized logistic regression model (Elastic Net, α = 0.5) using 10-fold cross-validation via the *cv.glmnet()* function from the glmnet package (v. 4.1.10) to select the optimal regularization parameter (lambda.min). Genes with non-zero coefficients at lambda.min, excluding the intercept, were retained as predictive features. Model performance was evaluated on the test set using a confusion matrix, sensitivity, specificity, and precision. To further explore the data structure, hierarchical clustering was applied to all samples based on the expression of these selected genes. To assess the robustness of DEG identification, we compared the Elastic Net-selected genes with those identified using the Limma method, and only genes consistently detected by both approaches were considered reliable. Hierarchical clustering of these DEGs across SCTs is visualized in Suppl. Fig. 3, where genes highlighted in green represent those also identified by Limma, and samples highlighted in red correspond to solid tissue normal, whereas those in black represent primary solid tumor.

### 2.3 Validation with independent datasets

We further validated the commonly identified genes across SCTs using independent datasets to ensure the robustness and reliability of our findings. To this end, we used publicly available transcriptomic profiles from the NCBI Gene Expression Omnibus (GEO) dataset using the *GEOquery()* package (v. 2.70.0) in R [35], including BRCA (GSE42568: 104 tumors, 17 normal samples) [36], LUAD (GSE32863: 58 tumors, 58 normal samples) [37], COAD (GSE44076: 98 tumors, 50 normal samples) [38], and PRAD (GSE46602: 36 tumors, 14 normal samples) [39]. Raw expression values were log2-transformed to approximate normality, and quantile normalization was applied using the *normalizeQuantiles()* function in R to minimize technical variability. For missing or infinite values, we used the k-nearest neighbors imputation method [40] by using *impute.knn()* function from the impute package (v. 1.82.0) in R.

The DEGs were identified using the limma package by fitting linear models between tumour and normal groups. In this validation, we focused on the directionality of gene expression changes, whether upregulated or downregulated (rather than significance level), to capture consistent expression patterns across datasets. Additionally, we calculate the Pearson correlation (r) and the corresponding P-value between TCGA and GEO gene expression values for common DEGs to assess the reproducibility quantitatively, validate the biological relevance, and demonstrate the robustness of our observed gene expression patterns across independent datasets.

To further validate the identified hub genes across SCTs, we analyzed proteomics data to strengthen the reliability of our findings. However, a considerable portion of the data was either unavailable or not statistically significant when analyzed using UALCAN-2025 (*The University of Alabama at Birmingham Cancer Data Analysis Portal*), an online platform that integrates datasets from the *Clinical Proteomic Tumor Analysis Consortium* (CPTAC) and the *Human Protein Atlas* (HPA) [41]. Additionally, we validated the expression patterns of these hub genes in a larger patient cohort of SCTs using TNMplot (https://tnmplot.com/analysis/) [42], which integrates data from TCGA, Gene Expression Omnibus (GEO), Genotype-Tissue Expression (GTEx), and Therapeutically Applicable Research to Generate Effective Treatments (TARGET). The TNMplot includes 56,938 unique samples from various sources (as of 12–09–2025), with 15,648 normal, 40,442 tumors, and 848 metastatic tumor samples. We further cross-checked their expression profiles across 33 TCGA Pan-cancer types, confirming that the observed up- or downregulation patterns were consistent and reproducible across independent datasets and multiple cancer types.

### 2.4 Weighted gene correlation network analysis (WGCNA)

Traditional gene expression analysis identifies DEGs between tumor and normal datasets, treating each gene independently and overlooking the complex connections within the transcriptome. This limits its ability to identify key drivers of disease affected by diverse factors influencing gene expression profiles. In contrast to random networks, scale-free networks exhibit a notable abundance of highly connected nodes known as "hubs" [43], [44]. These hubs are central within their networks, reflecting specific functional roles. The WGCNA uses scale-free networks to identify gene relationships, detecting modules of highly correlated genes and key hub genes that contribute to phenotypic traits.

In this study, a scale-free network was built using gene expression profiles of DEGs with the WGCNA (v. 1.73) package, allowing the construction of a weighted correlation network from tumor and normal SCT datasets. Gene expression matrices were first transformed into similarity matrices using the Pearson correlation coefficient between gene pairs. These similarity matrices were then converted into adjacency matrices by applying a soft-thresholding power. The power values were selected as the lowest power at which the scale-free topology fit index (R²) reached 0.8, ensuring the network approximates a scale-free topology while maintaining a relatively high mean connectivity. This process emphasizes strong gene-gene connections while minimizing weak correlations. The adjacency matrix was transformed into a topological overlap measure (TOM) to evaluate the strength of gene interactions. Genes were then hierarchically clustered based on TOM values, and gene modules were identified using the *DynamicTreeCut* algorithm, and modules with high similarity scores were merged. Additionally, module eigengenes (MEs) represent the overall expression profiles of their respective modules, and module membership (MM) is defined as the correlation between each gene's expression and the corresponding ME. Furthermore, we checked the relationship between module genes and specific traits by analyzing the distribution of their correlation values.

### 2.5 Gene set enrichment analysis (GSEA)

All DEGs from SCTs were further analyzed using the GSEA of Reactome pathways performed with the *gsePathway()* function from the clusterProfiler package (v. 4.10.1) in R. We set the threshold for pathway selection with *P-value* cutoff < 0.05, minGSSize = 3, and maxGSSize = 500. All the pathways were mapped over the Reactome hierarchy map [45] obtained from the Reactome database (v. 92) (Pathways hierarchy relationship data). In this network map, each node represents a biological pathway, and edges illustrate hierarchical or functional relationships between them. Node size corresponds to the number of genes in the pathway (gene set size). In contrast, node color reflects the normalized enrichment score (NES), with red indicating positively enriched pathways and green indicating negatively enriched ones, which reflects both the direction and magnitude of enrichment. The border color of each node indicates its statistical significance. Darker borders mean stronger significance, while lighter borders mean weaker significance. This integrated visualization emphasizes both the strength and reliability of pathway enrichment, along with their biological interconnections.

### 2.6 PPI network analysis and hub gene identification

A PPI network was constructed using the common genes identified through Limma, ENM, and WGCNA analyses to investigate their topological roles within the global interactome. Topological properties of the network provide valuable insights into the strength and nature of PPIs, revealing key interaction patterns and network architecture among nodes (i.e., genes). To construct the PPI network, we integrated entries from BioGrid (v. 4.4.236) [46] and HINT (High-quality Interactomes; version 2024–06) [47] to create a comprehensive dataset of documented human-human (Homo sapiens) PPIs. Although HINT already compiles high-quality PPIs from eight interactome resources, we combined these datasets to create a more robust network and maximize interaction coverage for our genes of interest. Next, clustering was performed using the unsupervised MCL algorithm to identify functional clusters in the PPI network based on stochastic flow. Parameters were set with a degree cut-off of ≥ 3 to include well-connected nodes, 1000 iterations for stability, and an inflation parameter of ≥ 2 to control cluster granularity. This approach revealed key biological processes and pathways within the identified modules.

### 2.7 Over-representation analysis of functional modules

After identifying clusters in the PPI network, we performed functional pathway analysis for each cluster with hub genes and their partners using Enrichr (v. 2025), a web-based tool [48] that can be found at https://maayanlab.cloud/Enrichr/. This tool allows users to submit lists of genes (human or mouse) for comparison against various gene set libraries, such as MSigDB [49], KEGG [50], Elsevier pathway collection (EPC) and Reactome database [51]. We integrate all databases for pathway analysis to ensure comprehensive coverage, as some pathways may be present in one database but absent in others for the query gene set. This approach enhances the robustness of insights derived from the clusters. These databases are reliable due to their rigorous curation, comprehensive pathway coverage, and regular updates. We set the adjusted *P* < 0.05 and the minimum gene set size to ≥ 2.

### 2.8 Validation and diagnostic assessment of hub genes

The TNMplot (v. 2.0) platform [42] comprises 56,938 unique samples from various sources (as of 12–09–2025), including 15,648 normal samples, 40,442 tumor samples, and 848 metastatic tumor samples. This large cohort provides a comprehensive view of gene expression in normal and cancer tissues, and thus was used to validate the association of our identified gene set with disease. To assess the broader relevance of the hub genes, we quantified their mean expression in a cohort of tumors across SCTs. A Wilcoxon rank-sum test was used to identify statistically significant differences in expression (p < 0.05) between this aggregated tumor cohort and normal tissue.

To assess the diagnostic potential of the 26 hub genes, we performed a cancer-type–specific analysis using receiver operating characteristic (ROC) curves generated from TCGA transcriptomic data with the TCGAplot (v. 8.0.0) package in R [52]. This approach evaluates the gene set's ability to function as a molecular biomarker by distinguishing between tumor samples and normal tissues. The diagnostic accuracy of the up- and down-regulated gene sets was measured using the area under the curve (AUC). Following established criteria, we classified performance as *AUC > 0.9*: excellent, *AUC > 0.8*: good, and *AUC > 0.7*: potentially useful. This analysis represents an essential step in biomarker discovery, as gene sets with high diagnostic power (*e.g., AUC > 0.8*) may serve as promising candidates for further investigation. Furthermore, assessing the consistency of expression of hub genes associated with cancer metastasis across two different profiling technologies (GeneChip and RNA-seq) strengthens our findings. We again utilized TNMplot to perform additional comparative gene expression analysis and compare primary tumors with metastatic tumors in SCT tissues. Significance in the GeneChip analysis was determined by a Kruskal-Wallis *P-value* < 0.05 for hub genes within the SCTs. However, this threshold for significance was largely unmet in the RNA-seq data (except for BRCA). This analysis utilized GeneChip data due to its substantially larger sample size for metastatic cases compared to the RNA-seq cohort, providing greater power for expression profiling. The primary objective was to characterize expression patterns across normal tissue, primary tumors, and metastases, independent of statistical significance considerations. We further analyzed the expression of these hub gene sets across 33 TCGA Pan-Cancer types to evaluate their consistency, reveal common patterns of dysregulation, and explore their potential as universal biomarkers or therapeutic targets across cancers.

### 2.9 Correlation analysis of hub genes with immune-related genes (IRGs)

We performed a comprehensive correlation analysis between the 26 hub genes from the up- and down-regulated gene sets of SCTs and a wide range of IRGs, including immune checkpoint genes (ICGs), chemokines (*CCL1–CCL28, CX3CL1, CXCL1–CXCL17*), chemokine receptors (*CCR1–CCR10, CXCR1–CXCR6, XCR1, CX3CR1*), immune stimulators (*CD27, CD40LG, ICOS,* and *TNFRSF/TNFSF* family members), and immune inhibitors (*PDCD1/PDCD1LG2, CTLA4, LAG3, TIGIT, IDO1*). We further assessed the associations between immune cell infiltration patterns and immune scores across multiple cancer types. For these analyses, we also utilised TCGAplot (v. 8.0) to visualize and analyze the correlation between IRGs and hub genes across TCGA Pan-cancer datasets, aiming to uncover significant patterns and associations. This approach enabled us to characterize the immune landscape of SCTs and to explore how these hub genes may modulate immune responses.

### 2.10 Evaluation of the predictive effect of hub genes on cancer immunotherapy response

In this study, we used the ROC Plotter tool-2025 (https://rocplot.com/) [53] to identify and validate hub genes associated with immunotherapy response, combining them into a signature gene set based on their mean expression across a wide range of solid tumor types. These include non-small cell lung cancer/non-squamous lung cancer, colorectal cancer, and breast cancer, as well as other tumor tissues (e.g., melanoma, glioblastoma, gastric cancer, bladder). Since the ROC Plotter combines data from many cancer types, our results show the overall performance of the hub genes across cancers. The values reflect their predictive power across a wide range of tumors, not just in SCTs.

Predictive performance was assessed using the AUC from receiver ROC analysis, and statistical significance between responders and non-responders to immunotherapy was determined using a *P-value* < 0.05. Immune-checkpoint inhibitors (ICIs), a key class of immunotherapy, have revolutionized cancer treatment over the years, demonstrating significant efficacy in both solid tumors and hematological malignancies. ICIs can be broadly classified into three groups: (i) inhibitors of programmed cell death 1 (PD-1) expressed on immune cells such as lymphocytes and natural killer cells, (ii) inhibitors of programmed cell death ligand 1 (PD-L1), which is expressed on a wide range of somatic and tumor cells and (iii) inhibitors targeting cytotoxic T-lymphocyte–associated protein 4 (CTLA-4) on T cells.

### 2.11 Risk stratification and survival modeling using clinical features and hub gene expression

To validate the prognostic potential of the hub gene set, we performed overall survival (OS) and progression-free interval (PFI) analyses using the Gene Expression Profiling Interactive Analysis (GEPIA v. 3.0) [54] is an advanced, publicly accessible web server for comprehensive pan-cancer analysis of gene expression. Here, we used a combined set of 24 hub genes, including both up- and downregulated genes in SCTs *(Note: Two of the original 26 hub genes, FOLR1 and TNS4, were excluded from this analysis due to their cancer-specific expression patterns across SCTs).* We subsequently extended this analysis to determine whether the prognostic relevance of the identified hub gene sets extends beyond SCTs by performing a TCGA pan-cancer analysis across 33 human cancer types. The log-rank tests were performed, and survival plots were generated with corresponding Cox hazard ratios and 95 % confidence intervals (95 % CI). The combined expression profiles of the hub gene sets (both up- and down-regulated) were analyzed to assess their prognostic significance across SCTs. Additionally, these hub gene sets were evaluated for prognostic relevance in a pan-cancer cohort covering 33 different cancer types. The total number of patients combined from all four cancer types was approximately 594 in both the high-expression and low-expression groups. Similarly, when combining patients across all 33 cancer types, each expression group consisted of approximately 2323 patients. The group cutoff was determined using a quartile split. The high-expression group comprised the top 25 % of samples (above the 75th percentile), while the low-expression group consisted of the bottom 25 % (below the 25th percentile). Statistical significance between the groups was assessed using the log-rank test, with a *P-value* < 0.05 considered statistically significant.

Additionally, we utilized clinical and transcriptomic data from SCTs (tcga_data.rds, limma_res.rds) to evaluate the prognostic value of the predefined 26 hub genes in overall survival and risk stratification. We developed a multivariate Cox proportional hazards model that incorporates clinical variables (age categories, tumor stage, and gender) and gene expression values. Expression data (TPM, unstranded) were log2-transformed [log2(TPM + 1)] for normalization, and the selected gene expression profiles were merged with clinical data to construct the final cohort. Age was categorized into three clinically relevant groups: younger (<40 years), middle-aged (40–59 years), and older (≥60 years). Tumor stages III and IV were combined into an “Advanced” category to ensure sufficient sample size within each stratum. Cox proportional hazards models were fitted using the survival package (v. 3.8.3) to evaluate associations between clinical variables, gene expression, and overall survival. The proportional hazards assumption was verified using Schoenfeld residuals, with stratification applied for variables violating this assumption. The Kaplan-Meier (KM) survival curves were generated for age categories, gender, tumor stage, and risk groups using the ggsurvplot function in the survminer package (v. 0.5.1), with global log-rank tests performed to compare survival distributions. Individual risk scores were calculated from the multivariate Cox model using the predict() function (type = "risk"), and patients were stratified into “Low Risk (better prognosis)” and “High Risk (worse prognosis)” groups based on the median score. Model discrimination was evaluated with the concordance index (C-index), and bootstrap resampling (boot package_(v. 1.3.28.1); 1000 iterations) was performed to estimate optimism and compute the optimism-corrected C-index. Predictive accuracy at multiple time points was assessed using a time-dependent ROC analysis package (timeROC_v.0.4), with AUC calculated at the 25th, 50th, and 75th percentiles of observed event times. Hazard ratios and 95 % confidence intervals from the Cox models were visualized as forest plots using the *forestmodel* package (*v. 0.6.2*).

## 3 Results

### 3.1 Screening of differentially expressed genes (DEGs)

The RNA expression data were extracted from the TCGA database to compare DEGs between " Tumor" and "Normal" samples across SCTs. All DEGs were identified using criteria of absolute fold changes ≥ 1.5 and adjusted *P* < 0.05. The numbers of upregulated (U) and downregulated (D) genes in each cancer type were as follows: BRCA (*U = 1161, D = 2479*), LUAD (*U = 1635, D = 2453*), COAD (*U = 1548, D = 2827*), PRAD (*U = 620, D = 973*). We proceeded by selecting a set of 199 common genes (*U=41, D=158*) identified as significant by both the Limma and Elastic Net models, which showed similar expression patterns across SCTs.

To ensure the reliability and biological relevance of our findings, we performed a two-step comparison between the TCGA and independent GEO datasets of the SCTs. First, we compared the average gene expression levels between tumor and normal samples, which revealed highly consistent expression patterns with similar directional trends in both datasets. However, the absolute expression values vary in the GEO dataset, likely due to differences in platform (e.g., microarray vs. RNA-seq), normalization methods, or sample processing. However, this variation in scale does not affect the overall pattern of gene expression, as the directional trends remained highly consistent between datasets (TCGA and GEO).

This consistency supports the robustness of our findings and suggests that the observed transcriptional changes are reproducible across independent cohorts despite technical disparities. Second, we identified statistically significant (P-value < 0.001) positive correlations in the SCTs, indicating a conserved expression pattern across different platforms and patient cohorts. Together, these findings suggest that the 199 common genes across the SCTs and their expression patterns are unlikely to be artifacts of specific datasets but rather reflect potentially robust and biologically consistent signals, highlighting the reliability of our observations (Suppl. Data**-1A**). The transcriptomic and proteomic datasets reveal both consistent and divergent hub gene expression patterns across BRCA, LUAD, and COAD cancers (lack of significant hits in PRAD). Several genes, including *CAV1, CRYAB, DES, FHL1, MYL9, PYGM, RRM2, SORBS1,* and *SYNM,* show matching trends in both RNA and protein levels with strong statistical significance (P-value <1.0 ×10^−8^), indicating strong transcriptional regulation. However, some genes exhibit significant discrepancies between their mRNA and protein expression levels. *IQGAP3* is upregulated at the RNA level but downregulated at the protein level in all three cancers, suggesting post-transcriptional suppression. Similarly, *TPX2* shows RNA upregulation but inconsistent protein levels (DOWN in BRCA and COAD, UP only in LUAD), implying complex regulatory mechanisms. Genes like *FOLR1, MASP1*, and *TNS4* have missing proteomic data (N.A.) for COAD, which limits complete interpretation; however, they show partial alignment in BRCA and LUAD. Full details of the total and shared DEGs are provided in Suppl. Data**-1B-D.**

### 3.2 Gene set enrichment analysis (GSEA)

We performed GSEA to identify common pathways among the gene expression datasets of SCTs individually to gain a broader understanding of the underlying biological processes and to identify global trends in the data. The enrichment analysis revealed that cancer-related global gene sets were significantly positively enriched (NES ≥ 1.5) in pathways involved in cell cycle regulation, apoptosis, chromosome maintenance, transcription, translation, DNA damage checkpoints, DNA repair, intracellular transport, signal transduction, extracellular matrix organization, MHC class II antigen presentation, membrane trafficking, and cellular stress responses. These findings indicate extensive reprogramming of cellular processes in cancer to support uncontrolled proliferation, enhanced survival, adaptability to stress, and tumor progression [55], [56], [57], [58], [59], [60]. Conversely, pathways showing significant negative enrichment (NES ≤ –1.5), particularly shared between BRCA and COAD, included biological oxidations and ethanol oxidation, suggesting reduced detoxification of xenobiotics and aldehydes [61], [62]. Dysregulation of reversible hydration of CO₂ by CA IX indicated altered acid–base balance in tumor cells [63], [64]. Additional negatively enriched pathways involved impaired O₂/CO₂ exchange, altered adrenoceptor signaling, suppressed fat-soluble vitamin metabolism, chylomicron remodeling, and clathrin-independent endocytosis, collectively highlighting disrupted metabolic flexibility, altered lipid handling, enhanced invasiveness, and rewired tumor-microenvironment interactions [65], [66], [67], [68], [69], [70], [71], [72], [73]. In BRCA, LUAD, and PRAD, the shared pathways are primarily involved in vesicle trafficking, metabolism, signal transduction, and physiological functions. Their dysregulation indicates that cancer cells reprogram normal cellular processes to promote uncontrolled growth, survival, and stress adaptation [74], [75], [76]. Detailed pathway information is provided in the Suppl. Data**-1E.** The Reactome hierarchical pathway map for SCTs is presented in Suppl. Fig. 4, where each node represents a Reactome pathway and the edges denote functional relationships among them. The normalized enrichment score (NES) quantifies the extent to which a given gene set is consistently up- or down-regulated within a ranked list of genes. This normalization accounts for variations in gene set size, enabling comparison across pathways. A positive NES (red nodes) indicates that the genes in the pathway are predominantly up-regulated, whereas a negative NES (green) reflects pathways enriched with down-regulated genes.

### 3.3 Detection of co-expression modules by WGCNA

**Fig. 1:**
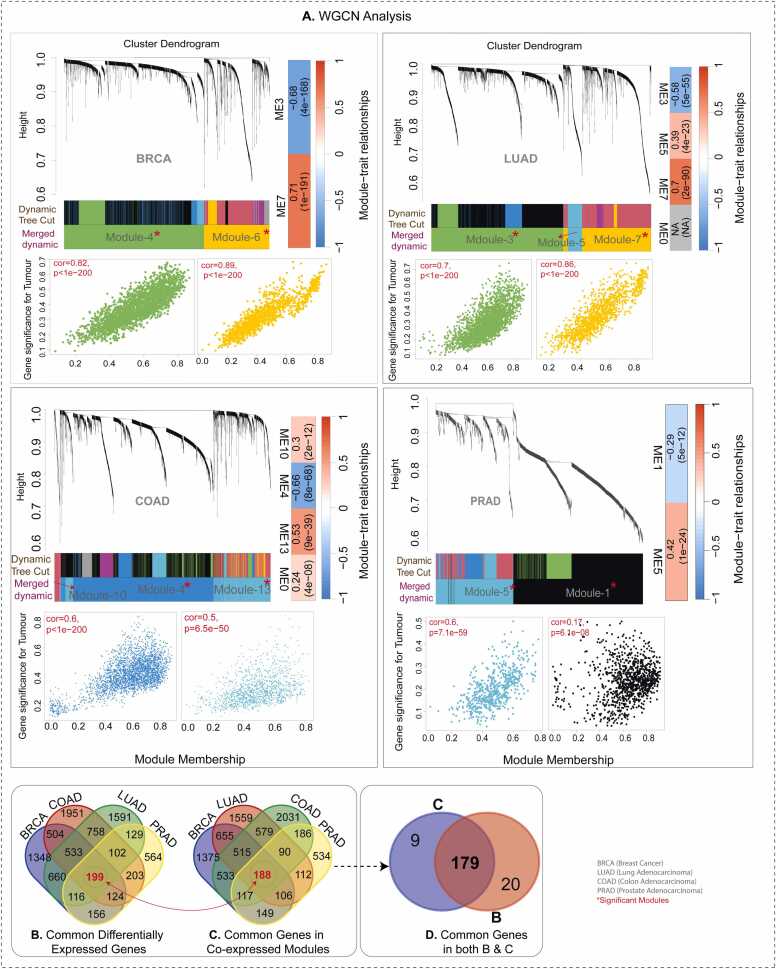
(A) Hierarchical clustering of genes using the 1-TOM matrix organizes co-expression network modules into a dendrogram for four cancer types (BRCA, LUAD, COAD, and PRAD). Each module is indicated by a distinct color. Scatterplots of gene significance (GS) for tumor samples versus module membership (MM) within each module show a strong correlation, indicating that hub genes in these modules are highly associated with tumor incidence across the four cancer types. (B–C) Venn diagrams depicting the overlap of differentially expressed genes (DEGs) among the four cancers identified using limma (B) and WGCNA (C). (D) Overlapping genes across modules from all cancer types, identified by integrating limma and WGCNA results.

All DEGs from SCTs were subjected to weighted gene co-expression network analysis (WGCNA) using the **WGCNA** package. Soft-thresholding power values for the datasets were selected based on a cutoff of R² = 0.8, ensuring that the resulting network approximates a scale-free topology (power value ≥ $R^{2}$). Module-trait relationships showed positive or negative correlations with tumors, with a high correlation between gene significance (GS) and module membership (MM), suggesting a strong association between key genes within the modules and the traits [77]. All modules across SCTs showed significant correlation with the tumor group, except for PRAD. All details about co-expressed modules are depicted in Fig. 1 **A**.

The WGCNA analysis identified significant co-expressed gene modules with a strong correlation (±0.5) to the phenotype (tumor vs. normal) and a *P*-value ≤ 0.05 across SCTs. Through comparative analysis of gene co-expression modules across SCTs, we identified 188 common genes, with 179 overlapping the 199 common DEGs detected by limma across multiple cancers (Fig. 1**B-D**). This intersection yielded a final set of 179 consensus genes derived from both methods. Among these, 157 genes (129 upregulated, 28 downregulated) exhibited consistent expression patterns across SCTs, while 23 genes demonstrated context-dependent flip regulation based on cancer type. For example, *AKR1B15, COL17A1, CRABP1*, and *ITPRID1* were downregulated in BRCA, COAD, and PRAD but upregulated in LUAD, highlighting their cancer type-specific regulatory behavior. Detailed information about each module and their key genes, along with MM and GS scores, is provided in Suppl. Data**-2**.

### 3.4 Protein-protein interaction (PPI) network and clustering analysis

We constructed a PPI network of 179 genes using the BioGRID and HINT databases, considering only physical and binary interactions to ensure high-confidence in direct protein–protein associations (comprising 2872 genes and 66,399 interactions). This approach excludes predicted or indirect interactions, thereby reducing false positives and emphasizing biologically meaningful interactions that are relevant to cellular function.

**Fig. 2:**
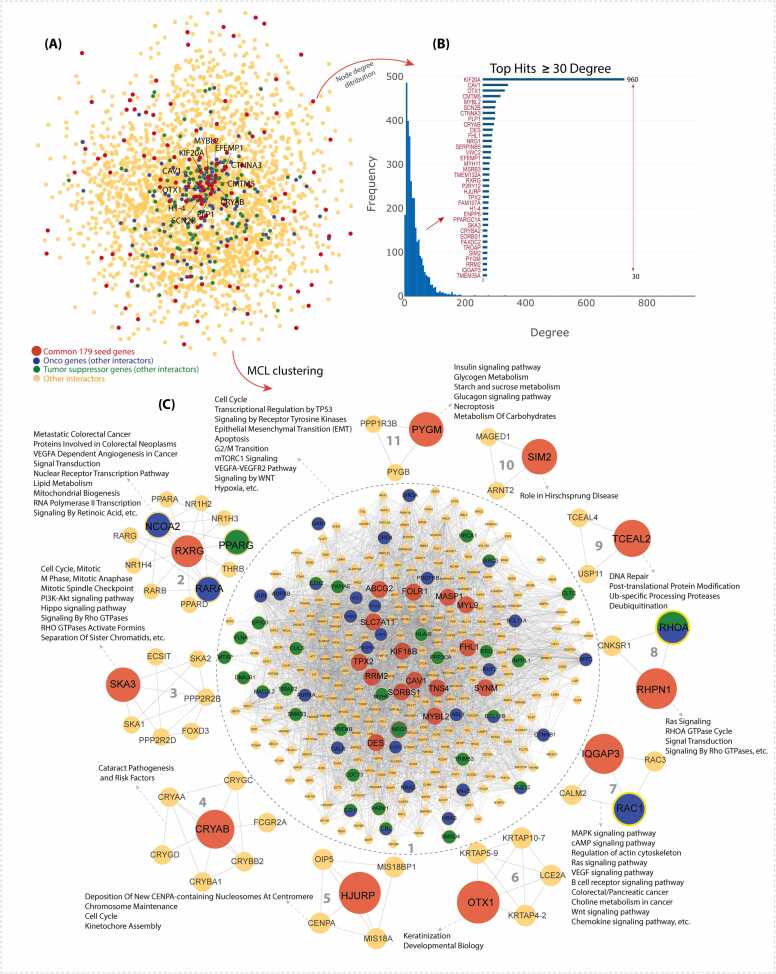
(A) PPI Network: The protein–protein interaction (PPI) network was constructed using 179 genes common to all cancer types (red nodes) along with their first-degree interaction partners (orange nodes), including oncogenes (blue) and tumor suppressor genes (green). Nodes are connected by edges, with hub genes located at the network’s center. (B) Node Degree Analysis: The bar graph depicts the distribution of node degrees, demonstrating the scale-free nature of the network, where a few genes exhibit high connectivity while most have low degrees. A separate bar plot highlights genes with a degree ≥ 30, identifying them as hub genes within the 179 common genes. (C) Functional Clustering: The PPI network was divided into 11 functional clusters using the Markov clustering (MCL) algorithm, each corresponding to distinct biological pathways.

We calculated the topological properties (degree, betweenness, closeness, and neighborhood connectivity) of the network to identify hub genes, which are located at the center of the network. These hub genes likely play key roles in maintaining the network's structure and function and may be crucial for understanding biological processes or diseases. The node-degree distribution suggests that the network follows a scale-free topology [78], a common feature of biological networks (Fig. 2 **A and B**). We identified several hub genes (including *KIF20A, CAV1, OTX1, CMTM5, MYBL2, SCN2B, CTNNA3, PLP1, CRYAB, DES, FHL1, NRG1, HJURP, TPX2, etc.*) within the PPI network, which strongly suggests that they may play critical roles in cancer-related biological networks.

Next, we applied MCL clustering to uncover functional clusters within the PPI network. We identified 11 functionally relevant clusters, each containing at least one hub gene from our list (Fig. 2 **C**). Clusters lacking hub genes were excluded from further analysis to prioritize modules with potential regulatory significance. These functional clusters enable a more detailed examination of complex biological systems, offering deeper insight into how genes interact to regulate cellular processes and pathways. Dysregulation of these pathways can contribute to the development of complex diseases, including cancers. Furthermore, pathway overrepresentation analysis was performed for each cluster, revealing that the majority were significantly enriched in cancer-related pathways, including ‘cell cycle and mitotic regulation’, ‘signaling pathways’, ‘angiogenesis’, ‘DNA repair and apoptosis’, and ‘metabolic pathways’. Additionally, we identified some inferred genes that interact with these hub genes and play significant roles in multiple cancers, underscoring the need for further investigation into their functions. The network topological properties and cluster pathways overrepresentation analysis are given in Suppl. Data**-3**.

### 3.5 Immune-correlation analysis based on hub genes

**Fig. 3:**
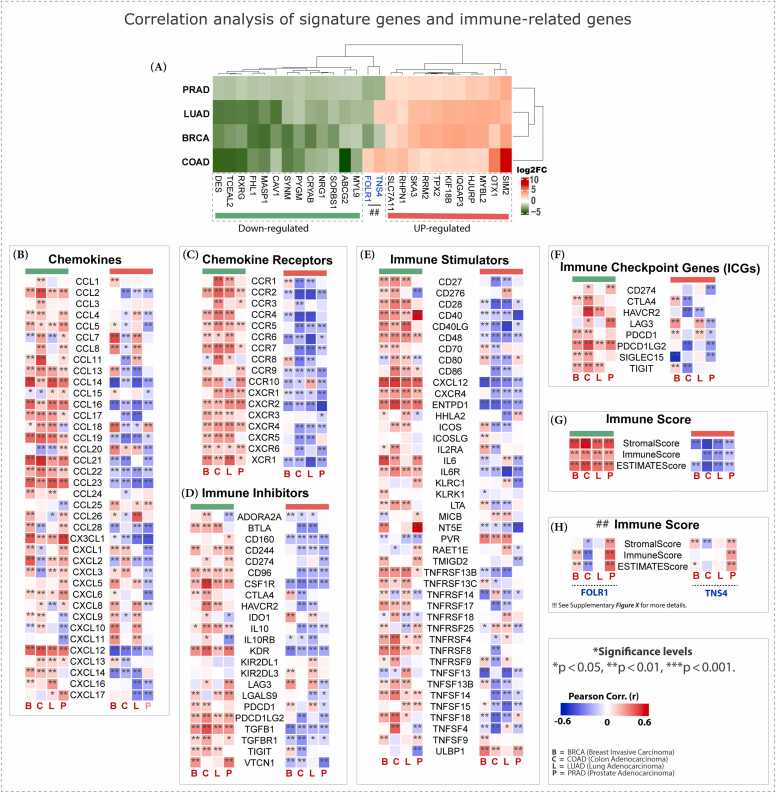
Correlation analysis between hub gene sets (up- and downregulated) and immune-related genes. (A) Reference heatmap showing expression patterns of 26 hub genes across SCTs. Correlation analyses with immune-related genes are shown for (B) chemokines, (C) chemokine receptors, (D) immune inhibitors, (E) immune stimulators, (F) immune checkpoints, and (G–H) overall immune scores. Positive correlations are indicated in red, negative correlations in blue, and statistical significance is denoted by asterisks (*).

Correlation analysis (P. corr) between the 26 hub genes (from both upregulated and downregulated SCT gene sets; Fig. 3**A**) and immune-related genes (IRGs), which include immune checkpoints, chemokines, chemokine receptors, and immune stimulators/inhibitors, across SCTs revealed a clear pattern: downregulated key genes (e.g., *MYL9, ABCG2, SORBS1, CRYAB, PYGM*) were positively correlated with IRGs, while upregulated key genes (e.g., *SIM2, OTX1, HJURP, IQGAP3, KIF18B*) showed negative correlations. For example, chemokine gene expression exhibited an inverse relationship with changes in gene expression in the SCTs. Specifically, numerous *CCL* and *CXCL* family members were found to be negatively correlated with upregulated gene sets. In contrast, a broader subset of *CCL* and *CXCL* genes exhibited positive correlations with downregulated gene sets, with only a few tissue-specific exceptions observed in specific cancers. Most correlations showed substantial and statistically significant associations (|r| > 0.5, *P*-value < 0.05), suggesting robust interrelationships between the IRGs and the identified hub genes (Fig. 3**B**).

A similar pattern was seen in other immune regulators. For the downregulated gene set, these IRGs showed strong positive correlations, while upregulated tumor gene sets showed strong negative correlations. This trend was also consistent with the total immune score and its components: the stromal score, immune score, and ESTIMATE score. This pattern was consistent across a wide range of immune components, including chemokine receptors (e.g., *CCR* and *CXCR* families; Fig. 3**C**), immune inhibitors (e.g., *CD274/PD-L1*, *IL10*, *CD96*, *TIGIT*; Fig. 3**D**), immune stimulators (e.g., *CD27, IL6, CD28, ICOS*; Fig. 3**E**), dedicated immune checkpoints (e.g., *CD274, LAG3, PDCD1*; Fig. 3**F**), and overall immune scores (Fig. 3**C-H**).

Additionally, some IRGs showed context-dependent associations. For example, among chemokines and their receptors, upregulated hub genes showed positive correlations with several members of the CCL and CXCL families (e.g., *CCL1, CCL4, CCL5, CCL20, CXCL1, CXCL5, CXCL8–11*), particularly in BRCA, LUAD, and COAD. In contrast, downregulated genes were negatively correlated with *CCL7, CCL15, CCL18, CCL20, CXCL1–3*, and receptors such as *CCR8* and *CCR10*. For immune stimulators, upregulated genes correlated positively with *TNFSF9*, *ULBP1*, *CD70, CD80, ICOS, ICOSLG, IL2RA, LTA*, and *PVR* in multiple cancers, whereas downregulated genes showed negative associations with *TNFSF13, MICB, TNFRSF18, CD276, HHLA2, PVR*, and *TNFRSF14*. Immune inhibitors were less frequently involved: a limited set of upregulated genes correlated positively with *CTLA4, TIGIT, LAG3, PDCD1*, and *IDO1*, while downregulated genes were negatively correlated with *ADORA2A, IDO1*, and *IL10RB*. Similarly, within the immune checkpoint genes, only a few positive correlations (*CTLA4, TIGIT, LAG3, PDCD1*) were observed, with most other checkpoints negatively associated with downregulated hub genes.

### 3.6 Predictive performance of hub genes in cancer immunotherapy response

**Fig. 4:**
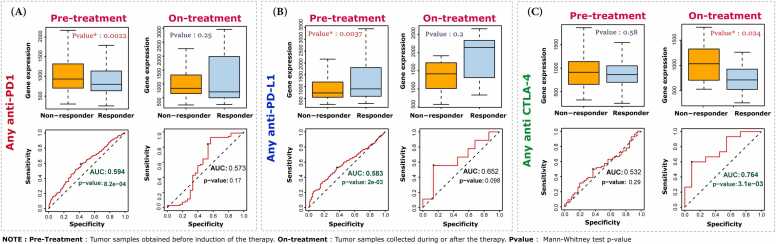
Predictive performance of the 26 hub gene set for treatment response. ROC curves assess the ability of the hub gene set to predict treatment sensitivity, while boxplots compare gene expression between responder and non-responder samples in a combined dataset of (A) anti-PD-1, (B) anti-PD-L1, and (C) anti-CTLA-4 pre- and on-treatment samples. Note: Pre-treatment refers to tumor samples obtained before therapy initiation, while on-treatment refers to samples collected during or after therapy. Statistical significance was evaluated using the Mann–Whitney test (p-value).

In pursuit of transcriptional biomarkers predictive of response to immune checkpoint inhibitors (ICIs), we analyzed the combined 26 hub genes (as a gene signature) for their predictive performance across anti-PD-1, anti-PD-L1, and anti-CTLA-4 therapies. This analysis was conducted on tumor tissues from lung, colorectal, and breast cancers, as well as other tumor types (e.g., melanoma, glioblastoma, gastric cancer, and bladder cancer), obtained both prior to starting the treatment (pre-treatment) and after treatment initiation (on-treatment) (Fig. 4). Our analysis revealed that the predictive power of the hub genes was significantly reduced during treatment with anti-PD-1 and anti-PD-L1 therapies. The hub genes demonstrated significant predictive performance in pre-treatment (pre-TRT) samples for both anti-PD-1 (AUC > 0.594; *P*-value < 8.2 × 10⁻⁴) and anti-PD-L1 (AUC > 0.583; *P*-value < 2.0 × 10⁻³) cohorts, but lost significance in on-treatment (on-TRT) samples (AUC = 0.573, *P*-value = 0.17 and AUC = 0.652, *P*-value = 0.098, respectively). In contrast, under anti-CTLA4 therapy, a striking pattern emerged, with the hub genes shifting from weak predictors in pre-TRT samples (AUC = 0.532, *P*-value = 0.58) to strong biomarkers in on-TRT samples (AUC = 0.764, *P*-value = 3.1 × 10⁻³).

### 3.7 Survival analysis (univariable)

**Fig. 5:**
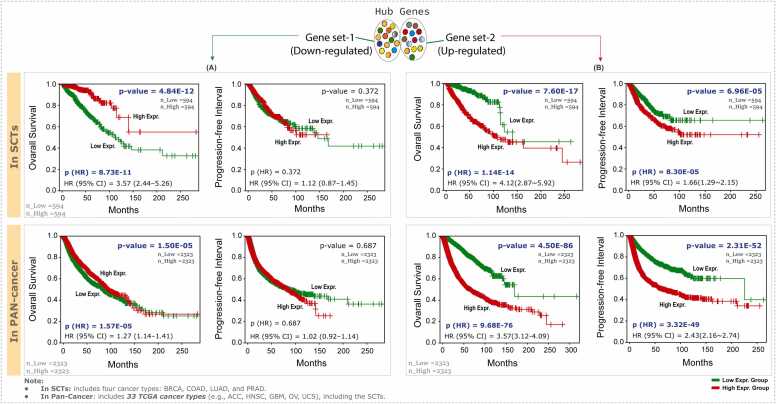
Kaplan–Meier survival analysis of the hub gene sets. Survival curves are shown for (A) the downregulated and (B) the upregulated hub gene sets, illustrating overall survival (OS) and progression-free interval (PFI) across SCTs and 33 cancer types (Pan-Cancer). Patients were stratified into high- and low-expression groups based on quartile cutoffs, represented in red and green, respectively. Hazard ratios (HRs) with 95 % confidence intervals (CIs) were estimated using the Cox proportional hazards model. The x-axis represents survival time (months), and the y-axis indicates survival probability.

The KM survival analyses were conducted to assess the prognostic relevance of hub gene sets in SCTs and across 33 cancer types, with *P*-values reported as p(LR) for the log-rank test and p(HR) for the hazard ratio, along with 95 % confidence intervals (CI). For the downregulated gene set, patients with low expression group exhibited significantly worse overall survival in SCTs compared to those with high expression (p(LR) = 4.84 × 10⁻¹ ², HR [95 % CI] = 3.57, p(HR) = 8.73 × 10⁻¹ ¹). Similar trends were observed in pan-cancer cohorts, where low expression also corresponded to reduced overall survival (p(LR) = 1.50 × 10⁻⁵, HR [95 % CI] = 1.27, p(HR) = 1.57 × 10⁻⁵). In contrast, for the PFI, the downregulated hub genes did not show a significant association in either SCTs or pan-cancer datasets (Fig. 5**A**).

For the upregulated gene set, patients with high expression group exhibited significantly reduced overall survival in both SCTs and pan-cancer cohorts. This association was supported by highly significant statistics for SCTs (p(LR) = 7.60 × 10⁻¹⁷, HR [95 % CI] = 4.12, p(HR) = 1.14 × 10⁻¹⁴) and for pan-cancer datasets (p(LR) = 4.50 × 10⁻⁸⁶, HR [95 % CI] = 3.57, p(HR) = 9.68 × 10⁻⁷⁶). For PFI, the high-expression group of the upregulated gene set was significantly associated with poorer PFI, indicating that the high-expression group of this gene set predicts a faster recurrence or progression of the cancer in both SCTs and pan-cancer cohorts. This correlation was confirmed by strong statistical significance in SCTs (p(LR) = 6.96 × 10⁻⁵, HR [95 % CI] = 1.66, p(HR) = 8.30 × 10⁻⁵) and pan-cancer datasets (p(LR) = 2.31 × 10⁻⁵², HR [95 % CI] = 2.43, p(HR) = 3.32 × 10⁻⁴⁹) (Fig. 5**B**).

### 3.8 Multifactorial prognostic analysis in the SCTs

**Fig. 6:**
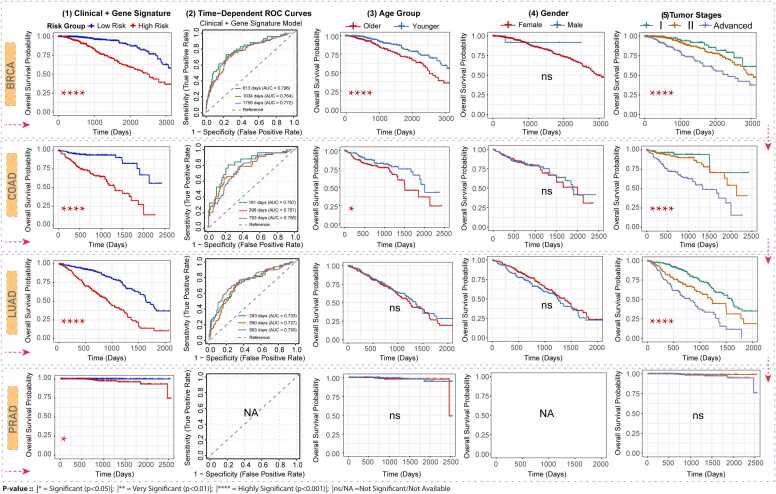

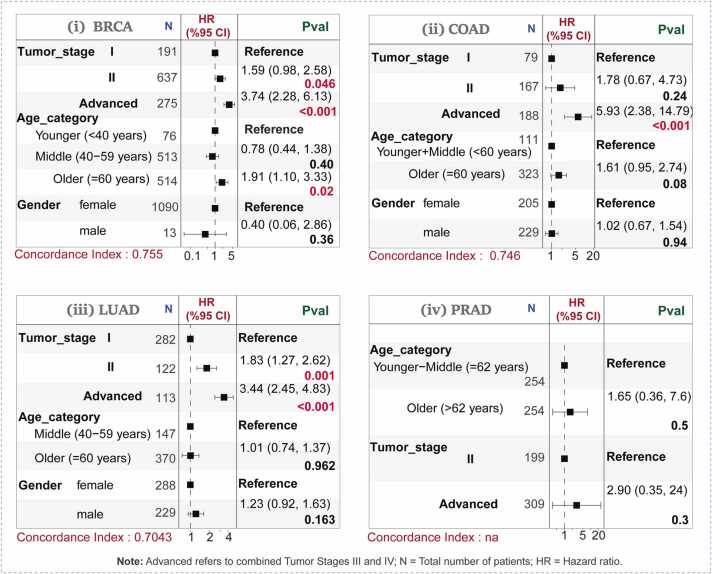
(A): A combined clinical–genomic risk model integrating clinical factors with hub genes stratified patients into low- and high-risk groups. The KM analysis revealed highly significant survival differences across all four independent cohorts (log-rank *P* < 0.001). *(1)* KM curves were plotted to compare outcomes between risk groups (high vs. low) for each cancer type. *(2)* Time-dependent ROC analysis demonstrated that the combined clinical and gene-based prognostic model exhibited strong predictive performance, with AUCs plotted at three time intervals, each represented by a different color. *(3–5)* Separate KM curves were generated for age, gender, and tumor stage to assess their individual impact on survival. For PRAD, gender information was not available. (B): Multivariate Cox regression models (forest plots) provided detailed risk assessments for clinical variables across SCTs. (i) In BRCA, tumor stage emerged as the strongest predictor, showing a stepwise increase in risk with advancing tumour stages. At the same time, older age (≥60 years) was also associated with poorer outcomes, while gender was not a significant factor. (ii) In COAD, advanced tumor stages similarly represented the major risk factor, whereas age and gender showed no significant effects. (iii) In LUAD, increasing tumor stage was also strongly associated with worse survival, while age and gender were non-significant. *(iv)* In contrast, within the PRAD cohort, none of the clinical variables, including age and tumor stage, showed any significant association with survival.

A combined clinical–genomic risk model that integrates clinical variables with the identified hub genes effectively stratified patients into low- and high-risk groups. KM analysis demonstrated highly significant survival differences across all four independent cohorts (p(LR) < 0.05; **Fig. 6A1**), confirming the model’s strong and consistent prognostic performance across SCts.

The time-dependent ROC analysis demonstrated the model's good to excellent predictive accuracy across multiple time points, with AUC values predominantly above 0.70 (**Fig. 6A2**). This was consistent with the overall model performance, as measured by the C-index (BRCA: 0.755; COAD: 0.746; LUAD: 0.704). Specifically, the AUCs for BRCA were 0.796, 0.764, and 0.772 at 613, 1034, and 1759 days, respectively. Similarly strong performance was observed in COAD (AUCs: 0.797, 0.767, 0.756) and LUAD (AUCs: 0.733, 0.727, 0.750) at 293, 590, and 993 days, respectively. A supporting forest plot confirmed that both clinical and molecular covariates contributed to this robust prognostic stratification, underscoring the value of the integrated model, particularly in the BRCA, COAD, and LUAD cohorts.

The prognostic analysis of demographic factors revealed distinct patterns. Age was a significant factor for BRCA and COAD patients, with older age being associated with poorer survival in both BRCA (p(LR) < 0.001) and COAD (p(LR) < 0.05), but not in LUAD and PRAD (**Fig. 6A3**). In contrast, gender showed no significant association with survival outcomes across SCTs, indicating it is not a major prognostic determinant across these cancer types (**Fig. 6A4**).

We found that tumor stage is a strong and reliable predictor of patient survival across SCTs. In the BRCA, COAD, and LUAD cohorts, the highly significant KM results (p(LR) < 0.0001) show that as tumor stage progresses from early to advanced, survival outcomes worsen in a consistent and statistically robust manner. In other words, patients with higher-stage tumors have significantly lower survival probabilities, confirming the prognostic importance of tumor stage in these cancers (**Fig. 6A5**).

Multivariate Cox regression analysis, visualized using forest plots, provided detailed risk assessments for each cancer type (Fig. 6**B**). (i) BRCA: Tumor stage was the strongest prognostic factor. Risk increased significantly from Stage I to Stage II (HR = 1.59, 95 % CI: 0.98–2.58, P = 0.046) and was substantially higher for combined Stages III/IV (HR = 3.74, 95 % CI: 2.28–6.13, P < 0.001), demonstrating a clear stepwise risk escalation. Older age (≥ 60 years) was also a significant risk factor (HR = 1.91, 95 % CI: 1.10–3.33, P = 0.02), while gender was not significant. The overall model was highly significant with good predictive accuracy (C-index = 0.755). (ii) COAD: Advanced stages (III & IV) constituted the most significant risk factor, showing a markedly increased hazard compared to Stages I-II (HR = 5.93, 95 % CI: 2.38–14.79, P < 0.001). Neither age nor gender was a significant predictor. The model demonstrated good discriminative performance (C-index = 0.746). (iii) LUAD: Advancing tumor stage was a powerful predictor of poor survival. The hazard significantly increased for Stage II (HR = 1.83, 95 % CI: 1.27–2.62, P < 0.001) and more than tripled for combined Stages III/IV (HR = 3.44, 95 % CI: 2.45–4.83, P < 0.001) relative to Stage I. Age and gender were not significant. The model was strongly significant with good predictive performance (C-index = 0.70). (iv) PRAD: In the PRAD cohort, none of the clinical variables, including age and tumor stage, showed a significant association with survival.

### 3.9 Validation of key gene expression patterns across a large cohort

**Fig. 7:**
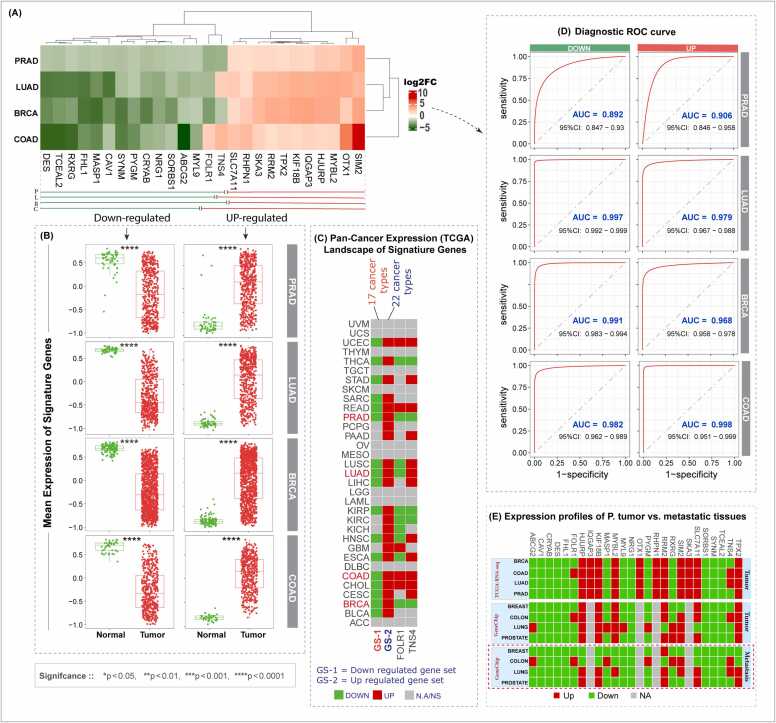
Validation and diagnostic assessment of hub genes. (A) Heatmap depicting expression patterns of 26 hub genes across SCTs. (B) Mean expression of hub genes in large tumor cohorts from BRCA, COAD, LUAD, and PRAD within the TNMplot dataset. (C) Expression consistency of the 26 hub genes across 33 TCGA Pan-Cancer datasets: downregulated gene set (GS-1; green) was suppressed in > 17 cancers, while upregulated gene set (GS-2; red) was overexpressed in up to 22 cancers; Two genes, *FOLR1* and *TNS4*, showed context-dependent expression across various cancers. (D) Diagnostic accuracy of up- and downregulated gene sets assessed by Area Under the Curve (AUC), classified as: AUC > 0.9, excellent; AUC > 0.8, good; AUC > 0.7, potentially useful. (E) Comparative analysis of hub gene expression in primary versus metastatic tumors across SCTs using two profiling platforms (GeneChip and RNA-seq).

Next, we validated the 26 hub genes using the TNMplot database, which contains nearly 57,000 clinical samples, including over 40,000 tumors, more than 800 metastatic samples, and over 15,600 normal tissues. The TNMplot analysis showed that the gene-expression patterns observed in TCGA were highly consistent across SCTs in this large multi-cohort dataset. This successful external validation confirms the robustness and broad applicability of these hub genes across diverse patient populations. As shown in the Suppl. Fig. 5, the expression levels of these hub genes were compared between normal and tumor samples. The box plots illustrate this data, with the Y-axis representing gene expression values and the X-axis representing the sample groups (tumor vs. normal). A statistically significant *P*-value is shown in the upper right corner of each plot. Analysis of both individual genes and their mean expression across the SCT cohort demonstrated strong and consistent differences between tumor and normal tissues. Both the upregulated and downregulated gene sets showed highly significant and coherent expression patterns (P < 0.0001) (Fig. 7**A–B**). To assess the broader significance of the identified hub gene sets (both up- and down-regulated), we examined their expression profiles across 33 TCGA pan-cancer types. This analysis evaluated the consistency of their dysregulation, identified shared expression patterns, and explored their potential as universal biomarkers or therapeutic targets. However, while differential expression provides an important initial indication, it does not confirm functional relevance. Therefore, experimental validation remains essential to establish their causal roles and clinical applicability. As shown in the Suppl. Fig. 6, the downregulated gene set (e.g., *MYL9*, *ABCG2*, *SORBS1*) was consistently suppressed in 17 cancer types, suggesting a potential tumor-suppressive-like role and involvement in shared oncogenic processes. In contrast, the upregulated gene set (e.g., *SIM2*, *MYBL2*, *HJURP*) was overexpressed in up to 22 cancers, indicating an oncogenic-like role and participation in common tumor-promoting pathways. The consistent dysregulation of these genes across multiple malignancies suggests that they are not tissue-specific but may represent a conserved mechanism shared among diverse cancer types, supporting their potential as broad-spectrum biomarkers and therapeutic targets. Interestingly, two of the 26 hub genes, *FOLR1* and *TNS4*, displayed context-dependent expression patterns across SCTs (Fig. 7**C**). However, experimental validation in relevant in vitro and in vivo models, as well as analyses in larger, independent cohorts, are needed to confirm their functional roles and clinical applicability.

Furthermore, we verified whether hub gene expression was consistent at the protein level by analyzing proteomics data across SCTs (except PRAD, for which data were unavailable) to strengthen the reliability of our findings (details are provided in Suppl. data 1B). Nine genes, including *CAV1, CRYAB, DES, FHL1, MASP1, MYL9, PYGM, SORBS1,* and *SYNM*, were consistently found to be downregulated at both the transcript and protein levels in BRCA, LUAD, and COAD, suggesting a role as tumor suppressors. In contrast, RRM2 was uniformly upregulated at both mRNA and protein levels, supporting its function as an oncogene. Two genes, *IQGAP3* and *TPX2*, were upregulated at the mRNA level but showed protein-level downregulation (*IQGAP3* in all cancers; *TPX2* in BRCA and COAD, up in LUAD), indicating post-transcriptional repression. A gene TNS4 showed context-dependent regulation because mRNA was down in BRCA but up in LUAD and COAD, while protein was down in BRCA and LUAD, suggesting tissue-specific post-transcriptional inhibition. Proteomic data for *FOLR1, MASP1*, and *TNS4* in COAD were unavailable, and 12 hub genes (*HJURP, KIF18B, MYBL2, RHPN1, SKA3, SLC7A11, ABCG2, NRG1, OTX1, RXRG, SIM2*, and *TCEAL2*) were either missing or not statistically significant across SCTs in the UALCAN (CPTAC and HPA) datasets.

We further evaluated the diagnostic potential of both hub gene sets using ROC curve analysis across SCTs. Both sets demonstrated exceptional performance in distinguishing tumor from normal tissue, with AUC values ranging from 0.892 to 0.998 (Fig. 7**D**), underscoring their strong promise as potential biomarkers. Additionally, analysis of hub gene expression patterns across SCTs revealed both conserved and cancer type–specific trends, which may be linked to tumorigenesis and metastatic processes (Fig. 7**E**). This analysis was carried out using RNA-Seq data for primary tumor samples (as metastatic sample data for SCTs were either very limited or unavailable), along with GeneChip data for both primary tumor and metastatic samples. Specifically, we compared primary tumor expression profiles using TCGA (RNA-Seq) and TNMplot (RNA-Seq) datasets. For metastatic samples, we relied on GeneChip array data. This approach allows us to cross-check expression consistency in primary tumors across platforms, while still deriving an approximate metastatic expression profile from available GeneChip data. Ultimately, this approach highlights expression differences between primary and metastatic samples, regardless of the underlying technology used (RNA-Seq vs. GeneChip).

A strong dichotomy emerged: many genes were consistently downregulated in both primary and metastatic tumors such as *CAV1, CRYAB, FHL1, DES, MYL9, NRG1, SORBS1, SYNM, TCEAL2, MYBL2* (except in lung↑), *MASP1* (except colon/lung↑), *FOLR1* (except colon↑), *ABCG2* (except colon/lung↑), and *PYGM* (except colon/lung↑) suggesting they may act as cancer-suppressive genes. Conversely, several genes were consistently upregulated in metastasis, though with cancer-type specificity. For instance, *TPX2, SLC7A11, KIF18B,* and *HUJURP* were upregulated in lung and prostate metastases but downregulated in colon and breast. *RRM2* was upregulated in breast, lung, and prostate metastases, but downregulated in colon. Similarly, *TNS4* and *SIM2* were upregulated in colon and lung but downregulated in breast and prostate metastases. For a few genes (*RHPN1, OTX1, SKA3,* and *IQGAP3*), metastasis-specific data were unavailable; however, they were consistently upregulated in primary tumor tissues (Suppl. Data 1 **F**).

These results suggest that cancer progression involves both conserved and cancer-type–specific gene regulation. Genes consistently downregulated in metastasis likely function as tumor suppressors whose loss facilitates metastasis. In contrast, genes show selective upregulation depending on cancer type, indicating context-dependent oncogenic roles. The observed dichotomy highlights that while some molecular mechanisms of metastasis are shared across SCTs, others are cancer-specific. Although metastasis-specific data are limited, these findings still enhance our understanding of tumor biology and suggest potential biomarkers of cancer progression. With more comprehensive datasets in the future, these candidate genes may also help identify therapeutic targets tailored to specific cancer types and stages of metastasis.

## 4 Discussion

A major challenge in oncology is identifying robust molecular signatures that are consistent across different cancer types. Computational approaches address this by systematically mining large-scale transcriptomic data, enabling unbiased identification of shared and unique oncogenic mechanisms. Such in silico analyses provide a rapid, cost-effective strategy to discover potential biomarkers and therapeutic targets, which can then be prioritized for experimental and clinical validation. To leverage this potential, we conducted a pan-cancer analysis of four major human cancers: breast, lung, colon, and prostate, by using TCGA datasets. Our goal was to develop a common transcriptomic signature to support cancer diagnosis, prognosis, and therapy. While many pan-cancer and single-cancer studies have significantly advanced our understanding of key oncogenes and provided valuable resources for cancer research, they typically focus on individual genes or small, pre-defined gene sets, leaving broader systems-level interactions less explored, as they are not designed to capture the complex interplay between genes, identify non-obvious hub genes (also referred to as inferred genes) with central regulatory roles, or elucidate the cooperative activity of functional modules that drive disease progression. For example, *Rabby et al. (2025)* integrated bioinformatics and machine learning to identify hub genes (*CAV1, RRM2*) and cross-cohort biomarkers (*EDNRB, MME*) in lung cancer [79]. *Zhang et al. (2025)* demonstrated that *SLC7A11* is upregulated in hepatocellular carcinoma (HCC), predicting poor survival and shaping an immunosuppressive tumor microenvironment. *Su et al. (2022)* performed a pan-cancer analysis of *HJURP*, linking its overexpression to cell cycle and p53 pathway dysregulation. Similarly, *Zhu et al. (2024)* and *Jeleń et al. (2024)* explored *TPX2* and *ABCG2*, respectively, across specific tumors or pan-cancer contexts [80], [81], [82], [83]**.**

Our study is distinguished by a systematic and biologically insightful framework that integrates statistical analysis (limma), network-based methods (WGCNA and PPI), and rigorous stepwise validation. This multi-tiered strategy minimizes false discoveries while identifying robust, clinically actionable hub genes and uncovering shared regulatory mechanisms across cancers that are often overlooked. Using this approach, we identified 179 shared DEGs, which were organized into 11 functional clusters and 26 hub genes (including both up- and downregulated sets) with high network centrality, highlighting their key regulatory and biological roles in cancer progression. Most of these hub genes are directly implicated in various aspects of cancer development: some are tumor suppressors whose downregulation may drive tumorigenesis, while others are oncogenes whose upregulation contributes to tumor progression. Several hub genes are involved either directly or indirectly in cancer-related processes, as reported in previous studies. The known properties of the downregulated / upregulated gene sets are discussed below:

**1. Integrated Mechanisms of Downregulated Gene Set:** The coordinated downregulation of the set of 13 genes disrupts core cellular processes, enabling tumorigenesis through convergent mechanisms. One of the central themes is the loss of cell cycle control, exemplified by *FHL1*, which enforces G1/S and G2/M phase arrest. Experimental validation of the function of *FHL1* has been reported. In one study, *FHL1* expression was shown to be downregulated in over 90 % of lung cancer patients. The *FHL1* protein acted to inhibit the growth of cancer cells via arresting the cells at the G1/S and G2/M phases. Moreover, overexpression of FHL1 resulted in a dramatic suppression in the growth of A549 lung cancer cells in nude mice [84]. Another study reported that knockdown of *FHL1* promoted tumor growth in nude mice, reinforcing its role as a tumor suppressor [85].

Two of the genes in our set, namely *MASP1* and *PYGM*, are involved in the regulation of lipid and glycogen metabolism, respectively [86], [87]. However, to date, credible functional validation data exist only for *MASP1*. Thus, in agreement with our findings, a recent publication reported that the expression of *MASP1* was downregulated in multiple human cancers, including the four SCTs investigated in the current study [88]. Validation of the role of *MASP1* protein was also carried out in hepatocellular carcinoma (HCC) cell lines. Overexpression of *MASP1* through lentiviral transfection in HCC cell lines was shown to inhibit the proliferation, migration, and invasion of the cancer cells in *in vitro* cell culture assays and *in vivo* as xenografts in nude mice, thus establishing *MASP1* as a tumor suppressor protein [88]. The evidence to date suggests that *MASP1* may be involved in reprogramming of lipid metabolism with consequent effects on various immunological and genomic factors, in the tumor immune microenvironment.

*ABCG2*, an ATP-binding cassette transporter, shows consistently low expression across most cancers, including SCTs, suggesting a common mechanism of downregulation during carcinogenesis. Loss of *ABCG2* impairs drug efflux and redox regulation, disrupting protective mechanisms such as autophagy and ferroptosis, creating a high-stress environment that promotes DNA damage and genomic instability. Surviving cells adapt under selective pressure, driving the emergence of aggressive tumor phenotypes [89].

The *CRYAB, MYL9, and SORBS1* genes also act as tumor suppressors through involvement in different signaling pathways. When overexpressed, *CRYAB* suppresses tumor cancer progression by inhibiting E-cadherin cytoplasmic internalization and maintenance of β-catenin in the membrane. Functional validation of its role was demonstrated in a study using nasopharyngeal carcinoma cells, in which overexpression of *CRYAB* was shown to inhibit cancer cell progression and EMT [90]. The tumor suppressor function of the myosin light chain 9 (MYL9) protein was recently functionally validated in non-small-cell lung cancer. This protein was shown to inhibit cancer cell migration, invasion, and EMT by counteracting the role of the associated myosin 19 (MYO19) protein [91]. Furthermore, *SORBS1* suppresses cancer cell invasion and metastasis by inhibiting JNK/c-Jun signaling and filopodia formation. Its loss enhances metastatic potential and chemoresistance through reduced p53 activity [92].

Another gene *CAV1,* encodes a protein known as caveolin-1 (Cav1) that is associated with lipid rafts. Reports have assigned conflicting roles for *CAV1* in cancer, acting both as a tumor suppressor and as an oncogene, depending on the type and stage of cancer. Knockdown of Cav1 by siRNA in a human breast cancer cell line promoted its proliferation and invasiveness by enhancing the activity of large-conductance Ca²⁺-activated potassium channels [93]. Conversely, upregulation of Cav-1 protein suppressed the proliferation and invasion of breast cancer cells. Another study investigated the role of Cav-1 in the metastatic potential of murine triple-negative breast cancer cells [94]. Cav-1 knockout in these cells suppressed lung metastasis in a syngeneic mouse model, providing the first in vivo evidence of the role of Cav-1 in regulating the migration capacity of breast cancer cells.

Retinoid X receptor gamma (RXRg) is a nuclear receptor for retinoids that is reported to play a role in the growth and differentiation of normal and tumor cells. Its expression has been shown to be downregulated by epigenetic modification in non-small cell lung cancer and hence, *RXRG* is regarded as a tumor suppressor gene [95]. Similar findings have been reported for *RXRG* in breast cancer cells [96]. Nevertheless, direct validation of the functional consequences of disrupting or overexpressing this gene in different cancer cell types is still lacking. The gene *NRG1* appears to have a context-dependent function in different cancers. Expression of *NRG1* was shown to be downregulated in lung cancers; addition of exogenous NRG1 reduced cancer cell proliferation and migration, while its downregulation by siRNA promoted cell growth, migration, and invasion potential [97]. In sharp contrast, elevated expression of NRG1 was observed in esophageal squamous cell carcinoma, and this was associated with poor disease outcome [98]. Silencing NRG1 expression in tumor cells disrupts important signaling pathways, such as PI3K/AKT, and leads to inhibition of tumor cell growth and migration. For the remaining genes in this set, namely *TCEAL2*, *SYNM,* and *DES*, direct validation of their functional roles in different cancers remains to be done.

**2. Oncogenic Mechanisms of Upregulated Gene Set:** The overexpression of this gene set drives tumorigenesis by hijacking core cellular processes, with dominant themes emerging in cell cycle dysregulation, mitotic instability, and adaptive signaling. A primary oncogenic mechanism is the induction of genomic instability through aberrant mitosis. *TPX2* overexpression promotes centrosome amplification and aneuploidy by disrupting spindle assembly [99]. At the same time, *HJURP* amplifies chromosomal instability (CIN), leading to DNA missegregation that paradoxically activates pro-metastatic NF-κB signaling and an EMT phenotype [100]. This mitotic chaos is further enabled by *KIF18B* and *SKA3*, which drive proliferation by activating the Wnt/β-catenin pathway and hypoxic lipid metabolism, respectively [101], [102]. Beyond mitosis, these genes co-opt critical signaling hubs to enhance survival and proliferation. Among them, *IQGAP3* functions as a central signaling node, amplifying KRAS and TGFβ pathways to sustain intratumoral heterogeneity and promote an immunosuppressive microenvironment [103]. Similarly, *MYBL2* promotes cell cycle progression by transactivating targets, such as CDCA3, and interacting with FOXM1 to potentiate Wnt signaling, thereby further promoting tumor progression [104]. The transcription factor *OTX1* exemplifies pathway convergence, driving proliferation in various cancers through ERK, JAK/STAT, and Wnt/β-catenin activation [105]. A key oncogenic strategy involves enhancing cellular stress adaptation, with *RRM2* upregulated by oncogenic drivers such as KRAS and AKT to sustain DNA synthesis under stress conditions and promote therapy resistance [106]. Likewise, under hypoxic conditions, KDM4A SUMOylation induces *SLC7A11* upregulation, enabling cancer cells to evade ferroptosis [107]. The adaptive survival is complemented by *SIM2,* which directly accelerates the cell cycle by upregulating Cyclin D1 and CDK4 [108]. *RHPN1* acts as an adaptor linking ROPN1 to RhoA, promoting actin fiber formation and TNBC cell migration. Overexpression of ROPN1 is correlated with metastasis and a poor prognosis. Silencing RHPN1 blocks ROPN1-driven migration, making it a potential therapeutic target [109].

Two genes *FOLR1* and *TNS4* exhibit context-dependent roles in cancer, acting as oncogenes or tumor suppressors depending on tissue type and expression levels. *FOLR1* is frequently overexpressed in solid tumors, where it promotes proliferation, invasion, and poor prognosis; for instance, in colorectal cancer, *FOLR1* upregulation facilitates immune evasion via lactate-induced STAT1 lactylation to suppress MHC-I [110], [111]. Conversely, reducing *FOLR1* levels alters key apoptotic and MAPK pathway genes (BIRC3, caspases, FOS, DUSP1, PRKX, TNFRSF10A), sensitizing taxol-resistant nasopharyngeal carcinoma cells to chemotherapy [112]. Similarly, *TNS4 (CTEN**)*** functions as an oncogene in colorectal cancer and lung adenocarcinoma, where its overexpression promotes epithelial–mesenchymal transition, migration, invasion, aerobic glycolysis, and tumor growth through upregulation of glycolytic genes (HK2, LDHB, PKM, SLC2A1) and activation of β-catenin/c-Myc and EGFR signaling pathways, contributing to poor prognosis and drug resistance [113], [114]. In contrast, *TNS4* exhibits tumor-suppressor–like functions in breast and prostate cancers, where its downregulation promotes tumor growth and angiogenesis via the c-Cbl/β-catenin/VEGFA axis or disrupts normal growth-regulating pathways. [115], [116]. Thus, both *FOLR1* and *TNS4* have highly context-dependent mechanistic roles, where upregulation generally promotes tumor progression and survival, while downregulation can either restore therapeutic sensitivity (*FOLR1*) or remove growth restraint (*TNS4*).

The PPI network analysis suggests that tumorigenesis is driven not by single-gene alterations, but by the coordinated dysregulation of interconnected gene clusters. Pathway enrichment analysis of these clusters highlights the interconnected processes sustaining cancer progression, including proliferation and cell cycle regulation (e.g., *ABCG2, KIF18B, TPX2*), maintenance of genomic integrity (e.g., *HJURP, SKA3*), metabolic reprogramming and stress adaptation (e.g., *CRYAB, PYGM*), and immune evasion (e.g., *RXRG).* Key hub genes, such as *OTX1* and *IQGAP3*, promote invasion, metastasis, and angiogenesis, while context-dependent regulators, like *SIM2* and *RHPN1*, modulate tumor behavior in a tissue-specific manner. The consistent association of these genes with poor prognosis underscores their clinical relevance. Collectively, these findings illustrate a synergistic hijacking of cellular processes, providing a framework to understand tumor aggressiveness and identify potential therapeutic vulnerabilities in precision oncology.

In network biology, while hub genes often receive primary attention, inferred genes (bottleneck genes) may also often play crucial roles. This study also highlights the critical importance of inferred genes in network biology, which act as essential bridges that mediate the effects of hub genes and enable cross-talk between different functional modules. In this study, we identified key inferred genes, including *UBC, ANLN, KIF14, VIRMA, ESR1, CIT, MYC, CUL3, JUN, BIRC3, PRC1, KIF23, ECT2, PARK2*, and *TP53*, which show strong connectivity with hub genes. A cluster-by-cluster pathway enrichment analysis demonstrated that these inferred genes are involved in a wide array of crucial biological processes, including cell cycle and proliferation (regulation of gene expression, cell cycle, and centromere/kinetochore function), signaling pathways (nuclear receptor signaling, AMPK, PI3K-Akt, Rho GTPase, and VEGF signaling), cellular structure and specialization (myogenesis, keratinization, and actin cytoskeleton organization), metabolism & disease (insulin signaling, glycogen metabolism, cataract formation, and Hirschsprung disease). By integrating these findings, the study suggests that the interactions between hub and inferred genes may contribute to alterations in proliferative, signaling, and metabolic pathways. This integrated network effect suggests that hub genes may have therapeutic relevance, as targeting them could impact multiple interconnected pathways through their associations with inferred genes. Comprehensive information on hub and inferred genes, encompassing their pathways, interactions, and functional roles across the 11 clusters, is available in Suppl. Data 3–4.

Our comprehensive analysis suggests that the identified hub genes are potentially robust and may provide valuable insights into their possible roles in cancer diagnosis and progression. We further observed that both the up- and downregulated hub gene sets show promising diagnostic potential. The remarkably high AUC values (all > 0.89) across SCTs demonstrate a near-perfect ability to differentiate between tumor and normal tissues. This consistent performance underscores their utility as potent biomarkers for SCTs, suggesting they could form the basis of a reliable molecular diagnostic tool. Beyond diagnostics, our findings reveal the complex molecular dynamics underlying tumor progression and metastasis. We observed a pronounced dichotomy in the expression patterns of hub genes. A set of genes from the downregulated gene set, including *CAV1, CRYAB, FHL1, DES, MYL9, NRG1, and SORBS1* were also consistently downregulated in metastatic tissues across cancer types, strongly suggesting that they function as metastasis suppressors and that their loss is a frequent feature of advanced tumors. Conversely, the expression of putative oncogenes was highly context-dependent. For example, some hub genes, such as *TPX2, SLC7A11, KIF18B***,** and *HUJURP,* were upregulated in lung and prostate metastases but downregulated in colon and breast, while others, like *TNS4* and *SIM2*, showed the opposite pattern. These observations indicate that the molecular drivers of metastasis are governed by tissue-specific regulatory mechanisms rather than a universal transcriptional program. Overall, our results suggest that the identified hub genes not only have strong diagnostic utility but also capture key biological processes underlying tumor initiation and progression. Their reproducibility across datasets strengthens their translational potential. At the same time, the distinction between conserved and cancer-specific patterns in metastasis provides a framework for developing both broad-spectrum and precision-based therapeutic strategies.

We further analyzed the expression profiles of these 26 hub genes across 33 TCGA Pan-Cancer types. Our results revealed that genes in the downregulated gene set (e.g., *MYL9, ABCG2, SORBS1, NRG1, CRYAB*) were significantly suppressed in more than 17 cancer types that share the common outcome of losing tumor-suppressive functions, thereby promoting proliferation, invasion, metastasis, and therapy resistance in various cancers (including SCTs). Additionally, proteomic analysis across BRCA, COAD, and LUAD confirmed that nine hub genes (e.g., *ABCG2, CAV1, CRYAB, DES*) were consistently downregulated at both transcript and protein levels, consistent with the loss of their tumor-suppressive functions in cancer [117], [118], [119], while *RRM2* was upregulated, consistent with an oncogenic function. A few genes (*IQGAP3, TPX2, TNS4*) showed discordant mRNA and protein patterns, suggesting post-transcriptional or tissue-specific regulation [120], [121]. Data for 12 hub genes were unavailable or not significant in certain cancers, reflecting limitations in proteomic coverage. Overall, most transcriptomic alterations in hub genes (specifically down-regulated gene set) are mirrored at the protein level, reinforcing their potential roles in tumor suppression or oncogenesis.

The correlation analysis between hub genes and immune-related genes (IRGs) across cancers revealed distinct expression patterns. The downregulated gene subset (e.g., *MYL9, ABCG2, SORBS1, CRYAB*) was generally positively correlated with multiple IRGs, including chemokines, chemokine receptors, immune checkpoint genes, and immune stimulators, suggesting a hypothetical role in maintaining an immune-responsive microenvironment. Reduced expression of these genes in tumors may therefore be associated with impaired immune activation and enhanced immune evasion, consistent with previous reports [122], [123]. This model could be tested by examining whether experimental restoration of these genes' expression enhances immune cell recruitment and activation *in vitro* or *in vivo.*

Conversely, the upregulated gene subset (e.g., *SIM2, HJURP, IQGAP3, KIF18B*) showed predominantly negative correlations with many IRGs, potentially reflecting an association with the suppression of anti-tumor immune responses. These patterns support the hypothesis that overexpression of these genes could contribute to a less immunogenic or “immune-desert” microenvironment, potentially facilitating tumor growth and survival [124], [125]. Future work using knockdown models could determine if suppressing these hub genes is sufficient to reinstate an immune-responsive state.

Selective positive correlations between hub genes (from both up- and downregulated subsets) and certain IRGs, including chemokines (*CCL1, CCL4, CCL5, CCL20, CXCL1, CXCL5, CXCL8–11) and immune* stimulators (*TNFSF9, ULBP1, CD70, CD80, ICOS, ICOSLG, IL2RA, LTA, PVR*), were observed in specific cancers such as BRCA, LUAD, and COAD, suggesting context-dependent associations with immune regulation [126]. In contrast, immune inhibitory and checkpoint genes (e.g., *CTLA4, TIGIT, LAG3, PD-1, IDO1*) showed fewer correlations, indicating that tumors may employ selective immune evasion strategies rather than global immune suppression [127], [128].

It is worth noting that the downregulated gene subset showed positive correlations with stronger stromal, immune, and ESTIMATE scores across all STCs, indicating that their decreased expression is associated with heightened immune reactivity in the tumor tissue. Conversely, the upregulated gene subset correlated with reduced immune and stromal scores in the tumor microenvironment. It should be emphasized that the relationship between the immune system and the intratumoral environment is quite complex. For example, while the expression of the downregulated gene subset correlated strongly with heightened immune and stromal scores, it also correlated with higher expression levels of *PDCD1LG2*, the gene encoding the PD-L2 checkpoint ligand. PD-L2 usually interacts with PD-1 receptor on T cells and inhibits their activation. It is thus not surprising that reduced expression of the downregulated gene subset correlated with poor overall survival across more than 30 cancer types. Moreover, lowered expression of this gene subset also correlated with enhanced expression of many chemokines and chemokine receptors. A case in point is the coordinated enhancement in the expression of CXCL12, a chemokine known as stromal cell-derived factor 1 (SDF-1) and its receptor, the CXCR4 protein. The interaction between CXCL12 and CXCR4 promotes tumor growth and metastasis [129]. It is therefore not surprising that the low expression of the downregulated gene subset correlated with worse survival.

Overall, these correlative findings are hypothesis-generating, highlighting potential links between hub genes and the immune landscape across cancers. To move from association to mechanism, future studies employing single-cell transcriptomics to map these relationships at a cellular level, coupled with functional validation in model systems, will be essential to define any causative roles these hub genes play in modulating anti-tumor immunity.

Analysis of the correlation between the expression levels of the identified hub genes and prognosis following ICI therapy revealed the potential of this gene set as a predictor of response to anti-PD-1 and anti-PD-L1 treatments across more than 15 tumor types available in the ROC-Immunotherapy Plotter tool. Interestingly, the prognostic value of the gene set lost significance once the ICI treatment began (in the On-treatment tumors). It is well known that the administration of anti-PD-1 or anti-PD-L1 monoclonal antibodies leads to dramatic alterations in the tumor microenvironment, transforming “cold” non-inflamed tumors into “hot” T-cell-infiltrated tumors [130]. This transformation involves remodeling of the tumor stoma and vasculature [131], activation of immune and interferon response pathways [132], [133], reprogramming of exhausted and progenitor CD8^+^ T cells [134], [135], and induction of CD8^+^ T cell infiltration [136], [137]. Thus, the reduced predictive power of the identified gene set during anti-PD-1 and anti-PD-L1 therapy suggests a loss of pre-existing transcriptional distinctions between responders and non-responders. This could reflect adaptive resistance mechanisms or immune-driven transcriptional reprogramming during treatment.

Surprisingly, our analysis revealed that the prognostic value of the gene set in predicting response to immunotherapy is lost in patients receiving anti-CTLA-4 monoclonal antibody (mAb) treatment. While the reason for this difference is unknown, it is worth noting that the two types of ICI therapies, namely anti-PD1/PD-L1 and anti-CTLA-4, are thought to function quite differently within the tumor tissue. Anti-PD1/PD-L1 blockade primarily acts to reinvigorate intratumoral stem-like/progenitor-exhausted CD8^+^ T cells and IFN-g-driven inflammation [138], [139]. In contrast, CTLA-4 blockade acts by relieving the brakes in the priming phase during T cell priming, reprogramming T regulatory cells, and enhancing CD4^+^ T cell activation [140], [141]. Therefore, the gene set identified in our analysis appears to perform better in predicting the response to ICI therapies targeting CD8^+^ T cells and inflammatory cytokine gene signature within the tumor tissue than those targeting CD4^+^ T cells and T regulatory cells.

Intriguingly, the hub gene signature had a significant prognostic utility in predicting response to anti-CTLA-4 blockade in tumors already on-treatment. This underscores the distinctive features of both types of ICI therapies, with anti-CTLA-4 blockade acting early during the priming phase to influence CD4^+^ T cells, and anti-PD-1/PD-L1 acting later during the effector phase to promote CD8^+^ T cell activation, IFN-γ signaling, and chemokine-driven cellular infiltration. This distinction is highlighted by the superior response observed by combining the two ICI therapies in cancer patients [142], [143].

To determine whether the hub gene set influences patient survival, we observed that the downregulated gene set showed a significant association with poorer overall survival in SCTs and across 33 cancer types. This finding suggests that the loss of these genes may impair their protective or tumor-suppressive functions, thereby promoting more aggressive tumor behavior and unfavorable clinical outcomes [144]. Moreover, the consistent pattern across multiple cancer types (Pan-cancer) indicates that the cellular pathways regulated by this gene set are likely fundamental to maintaining tumor suppression in diverse tissues and organ systems [145]. It is noteworthy that while we observed a significant correlation with OS, the level of expression of the downregulated gene subset did not correlate with PFI. Although this may be perceived as contradictory, the results can be readily explained by various factors. For example, while the treatment may not affect progression to first recurrence, it could nevertheless improve patient OS by influencing response to subsequent treatmenttherapies or by slowing tumor growth after progression. Additionally, some treatments, especially those involving ICI blockade, show delayed beneficial effects through establishing durable, long-term survival and boosting subsequent OS. Thus, a given therapy may not delay cancer progression early on, but it can nevertheless ultimately lead to a significant enhancement in OS through improving patient survival after initial progression [146], [147]. Conversely, the upregulated gene subset demonstrated an even more pronounced prognostic capacity. High expression of the gene subset was not only linked to reduced OS but also to a significantly shorter PFI in both SCT-specific and pan-cancer analyses. This consistent association with PFI strongly suggests that these upregulated genes are actively involved in driving tumor recurrence and progression, making them promising oncogenic candidates and potential therapeutic targets.

On the other hand, the multifactorial risk model allowed robust stratification of patients into high-risk groups and low-risk groups (*P-value* < 0.05) across multiple cancer cohorts, demonstrating its universal prognostic utility and revealing significant survival differences between the two groups. Among clinical variables, (i) tumor stage consistently emerged as the strongest predictor of survival, showing an apparent stepwise increase in risk with advancing stage, (ii) whereas age and (iii) gender were generally not significant, except for younger age, which showed improved survival in BRCA. Overall, this study highlights both molecular and clinical determinants of prognosis in the SCTs, providing a framework for precise risk stratification and potential therapeutic targeting.

## 5 Conclusion

Overall, our integrative transcriptomic analysis identifies a set of hub genes that constitute a conserved oncogenic program influencing tumor progression, immune modulation, and patient prognosis across BRCA, COAD, LUAD, and PRAD. Although many of these hub genes have established roles in different cancers, our multi-layered validation reinforces their relevance and highlights their potential as cross-cancer potential biomarkers within these tumor types. We also uncovered novel inferred genes strongly associated with hub genes, representing promising targets for further investigation and potential therapeutic development. This work provides a robust platform for cross-cancer transcriptomic analysis and biomarker discovery, positioning these hub genes as key targets for future research in cross-cancer and immunotherapy. Despite these advances, limitations remain: reliance on public datasets may introduce selection biases, and focusing solely on gene expression overlooks other regulatory layers such as proteomics and epigenomics. Future studies should prioritize experimental validation to confirm the association between these hubs and patient outcomes, treatment responses, and resistance mechanisms, ultimately enabling more personalized and effective cancer management.

## CRediT authorship contribution statement

**Aftab Alam:** Writing – original draft, Formal analysis, Data curation, Conceptualization. **Basel K. Al-Ramadi:** Writing – review & editing, Writing – original draft, Supervision, Investigation, Funding acquisition, Formal analysis, Conceptualization. **Maria J. Fernandez-Cabezudo:** Writing – review & editing, Formal analysis. **Uday Kishore:** Writing – review & editing. **Rifat Hamoudi:** Writing – review & editing, Formal analysis. **Mohd Faizan Siddiqui:** Writing – review & editing, Formal analysis, Data curation.

## Declaration of generative AI and AI-assisted technologies in the manuscript preparation process

During the preparation of this work, Aftab used the Grammarly tool (Pro version) to remove grammatical errors and correct spelling and punctuation in the manuscript for smooth reading. After using this tool, he reviewed and edited the content as needed and takes full responsibility for the content of the published article.

## Declaration of Competing Interest

The authors declare that the research was conducted without any commercial or financial relationships that could be seen as a potential conflict of interest.

## Data Availability

All relevant supporting data files, including Cytoscape network files, supplementary datasets, and the R scripts used for data processing and analysis, are publicly accessible via the project's GitHub repository at https://github.com/aftab3866/Suppli_Data_2025
