# Supplementary material for: A systems biology approach to unveil shared therapeutic targets and pathological pathways across major human cancers
Source: Comput Struct Biotechnol J. 2025 Nov 29;27:5459–78. doi: 10.1016/j.csbj.2025.11.061 (PMC12720046; doi:10.1016/j.csbj.2025.11.061)
Supplement: Supplementary file 1 — Supplementary material [file mmc1.zip › Supplementary_figure-3.pdf]

A Systems Biology Approach to Unveil Shared Therapeutic Targets and Pathological Pathways Across Major Human Cancers

Aftab Alam[1], Mohd Faizan Siddiqui[2], Rifat Hamoudi[3,4,5], Uday Kishore[6,7], Maria Fernandez Cabezudo[7,8] and Basel K. Al-Ramadi[1,7,9]*

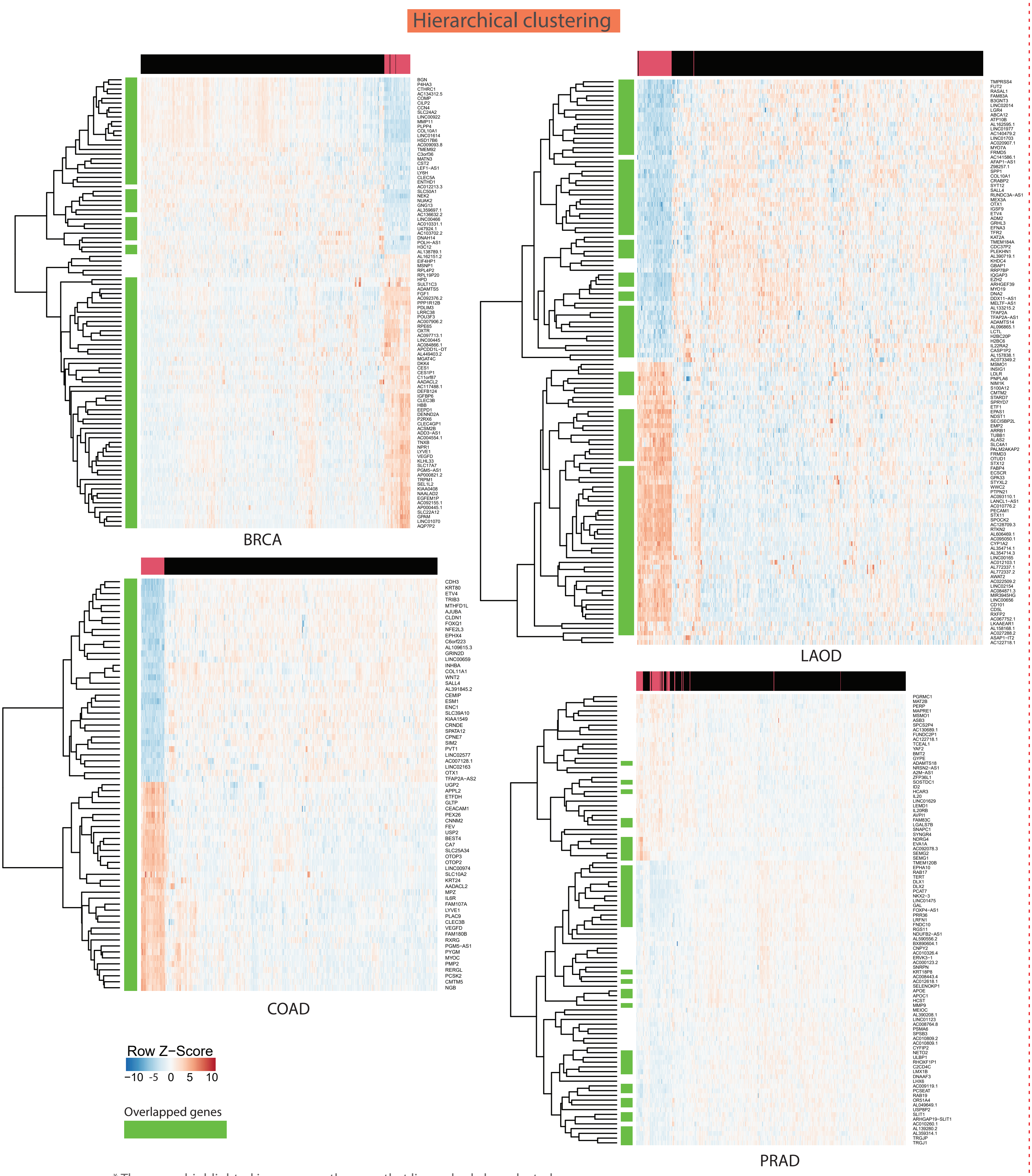

* The genes highlighted in green are the ones that limma had also selected.

* The samples highlighted in red are **Solid Tissue Normal**, the samples highlighted in black are **Primary solid Tumor**.
