# Supplementary material for: A systems biology approach to unveil shared therapeutic targets and pathological pathways across major human cancers
Source: Comput Struct Biotechnol J. 2025 Nov 29;27:5459–78. doi: 10.1016/j.csbj.2025.11.061 (PMC12720046; doi:10.1016/j.csbj.2025.11.061)
Supplement: Supplementary file 1 — Supplementary material [file mmc1.zip › Supplementary_figure-5.pdf]

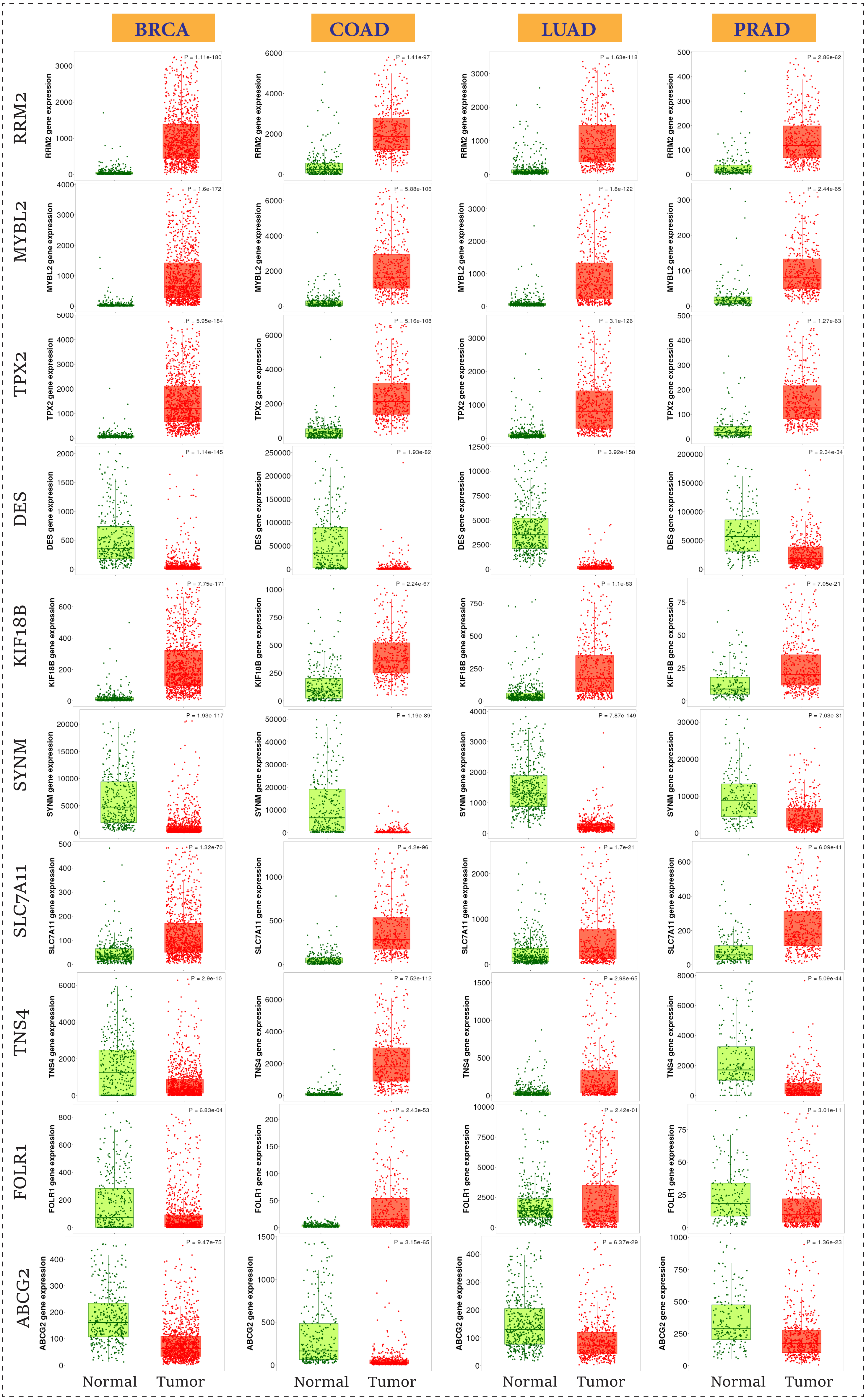

BRCA
COAD
LUAD
PRAD
RRM2
MYBL2
TPX2
DES
KIF18B
SYNM
SLC7A11
TNS4
FOLR1
ABCG2
RRM2 gene expression
MYBL2 gene expression
TPX2 gene expression
DES gene expression
KIF18B gene expression
SYNM gene expression
SLC7A11 gene expression
TNS4 gene expression
FOLR1 gene expression
ABCG2 gene expression
P = 1.11e-180
P = 1.41e-97
P = 1.63e-118
P = 2.86e-62
P = 1.6e-172
P = 5.88e-106
P = 1.8e-122
P = 2.44e-65
P = 5.95e-184
P = 5.16e-108
P = 3.1e-126
P = 1.27e-63
P = 1.14e-145
P = 1.93e-82
P = 3.92e-158
P = 2.34e-34
P = 7.75e-171
P = 2.24e-67
P = 1.1e-83
P = 7.05e-21
P = 1.93e-117
P = 1.19e-89
P = 7.87e-149
P = 7.03e-31
P = 1.32e-70
P = 4.2e-96
P = 1.7e-21
P = 6.09e-41
P = 2.9e-10
P = 7.52e-112
P = 2.98e-65
P = 5.09e-44
P = 6.83e-04
P = 2.43e-53
P = 2.42e-01
P = 3.01e-11
P = 9.47e-75
P = 3.15e-65
P = 6.37e-29
P = 1.36e-23
Normal
Tumor

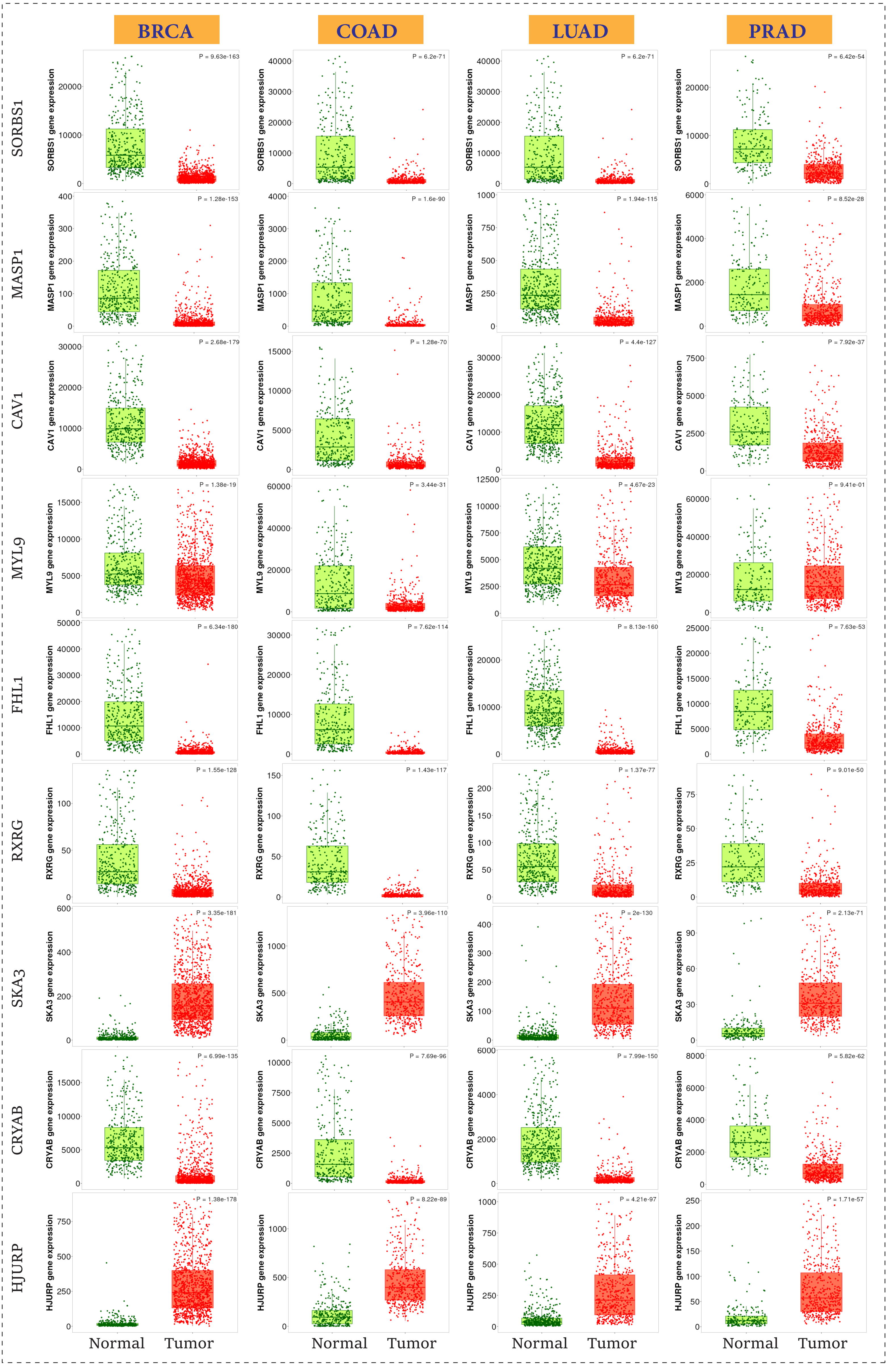

BRCA
COAD
LUAD
PRAD
SORBS1
MASP1
CAV1
MYL9
FHL1
RXRG
SKA3
CRYAB
HJURP
Normal
Tumor

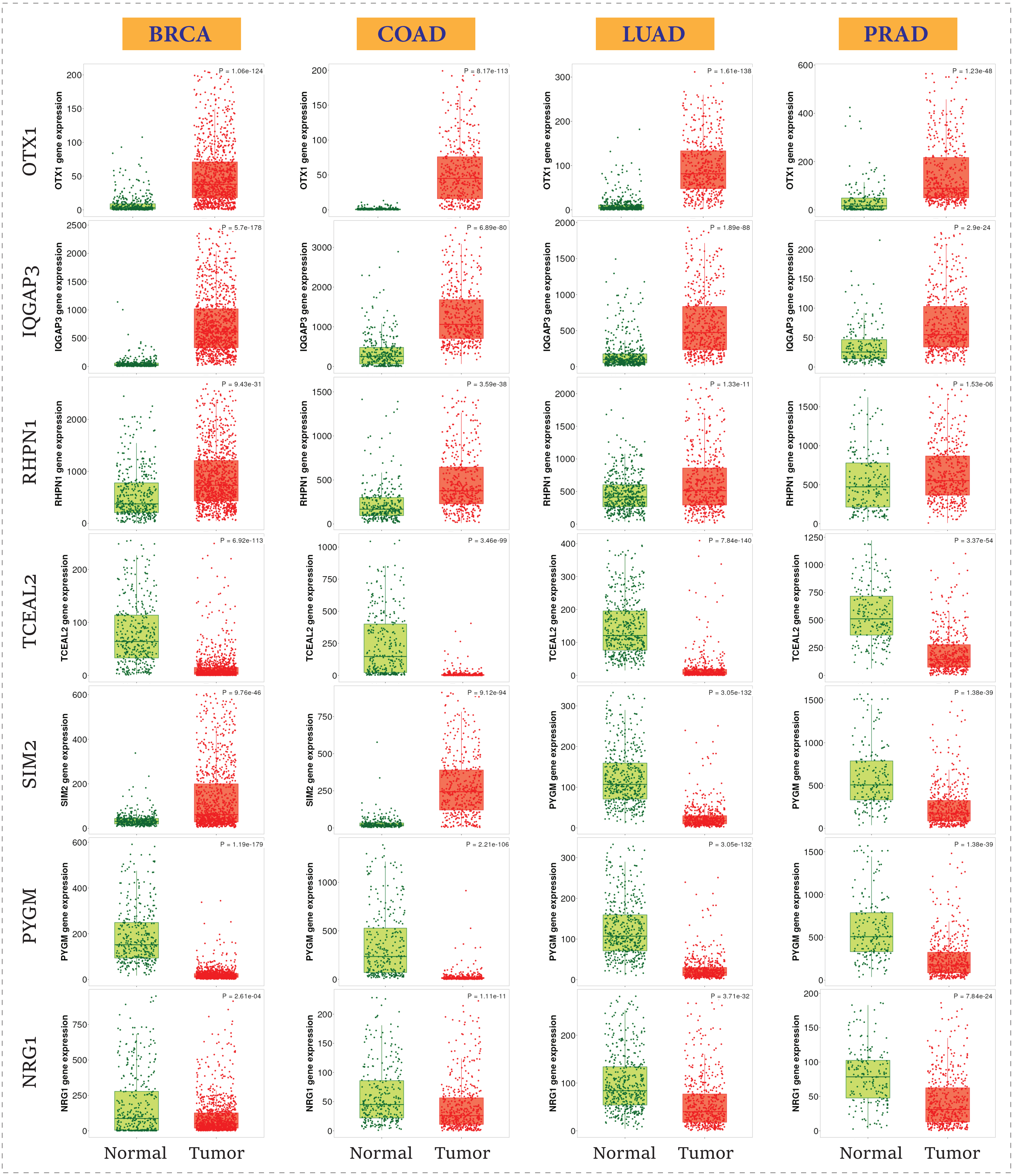

A Systems Biology Approach to Unveil Shared Therapeutic Targets and Pathological Pathways Across Major Human Cancers

Aftab Alam[1], Mohd Faizan Siddiqui[2], Rifat Hamoudi[3,4,5], Uday Kishore[6,7], Maria Fernandez Cabezudo[7,8] and Basel K. Al-Ramadi[1,7,9]*
