# Supplementary material for: A systems biology approach to unveil shared therapeutic targets and pathological pathways across major human cancers
Source: Comput Struct Biotechnol J. 2025 Nov 29;27:5459–78. doi: 10.1016/j.csbj.2025.11.061 (PMC12720046; doi:10.1016/j.csbj.2025.11.061)
Supplement: Supplementary file 1 — Supplementary material [file mmc1.zip › Suppli-4_data.docx]

**A Systems Biology Approach to Unveil Shared Therapeutic Targets and Pathological Pathways Across Major Human Cancers**

**Aftab Alam^1,^ Mohd Faizan Siddiqui^2^, Rifat Hamoudi^3,4,5^, Uday Kishore^6,7^, Maria Fernandez Cabezudo^7,8^ and Basel K. Al-Ramadi^1,7,9*^**


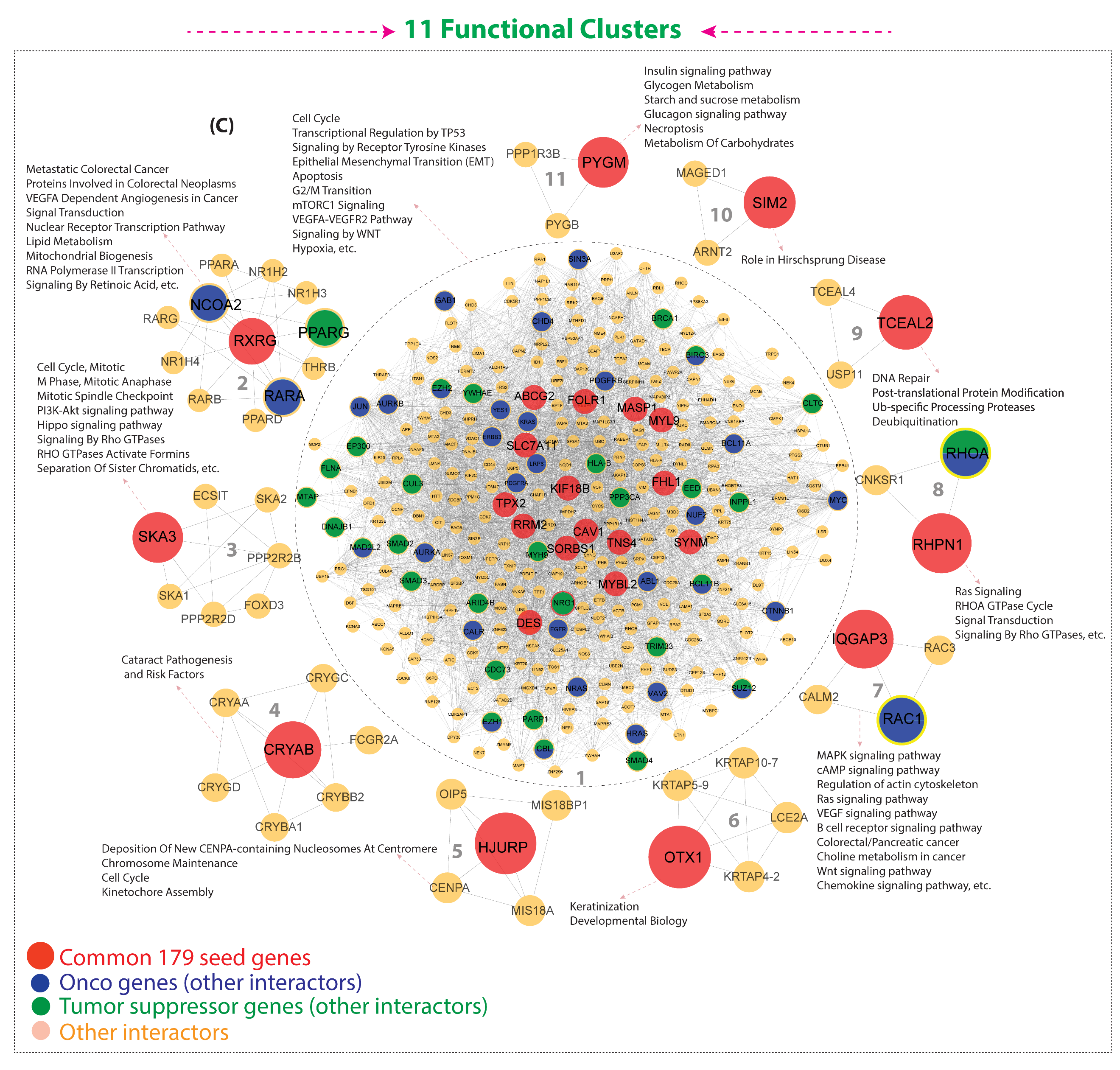


**Cluster-1**, the largest cluster, contains 15 hub genes (*ABCG2, KIF18B, SLC7A11, FOLR1, MASP1, MYL9, RRM2, MYBL2, TPX2, DES, SYNM, TNS4, CAV1, SORBS1*, and *FHL1*) that interact with 286 other genes, including 29 oncogenes and 28 tumor suppressor genes. This gene cluster is enriched in critical cancer-related pathways, including cell cycle, EMT, mTORC1 signaling, VEGFA-VEGFR2 pathway, Wnt signaling pathway, and hypoxia, suggesting a coordinated mechanism promoting tumor proliferation, survival, and metastasis. This synergistic network is regulated by central hub genes that coordinate critical cancer hallmarks. For instance, *KIF18B* and *TPX2* are both critical for mitotic spindle assembly and chromosome segregation during mitosis; their upregulation in numerous cancers promotes genomic instability and uncontrolled proliferation, and is linked to poor prognosis and therapy resistance [1–4].  Similarly, *RRM2*, the catalytic subunit of ribonucleotide reductase, is essential for DNA replication and repair. Its upregulation in cancers such as those of the kidney, lung, and liver is also linked to poor prognosis [5–7]. The oncogenic transcription factor *MYBL2* further fuels proliferation and has been shown to sustain an immunosuppressive tumor microenvironment by collaborating with CCL2 to recruit immunosuppressive macrophages, thereby promoting resistance to immunotherapy [8,9]. Concurrently, the cystine transporter *SLC7A11* supports survival under metabolic stress by enabling glutathione synthesis, which protects against oxidative stress-induced cell death and contributes to therapeutic resistance [10,11]. The collective function of these hub genes points to a synergistic network that drives aggressive tumorigenesis through complementary roles in promoting cell division, suppressing immune attack, and enhancing cellular survival.

**Cluster-2** comprises 12 genes centrally regulated by *RXRG*, a nuclear receptor that modulates retinoic acid signaling, apoptosis, and the epithelial-mesenchymal transition (EMT). The function of *RXRG* is highly context-dependent, exhibiting dual roles in oncogenesis: it can act as a tumor suppressor, as evidenced in breast cancer, or as an oncogene that promotes aggressive tumor growth in lung and thyroid cancers [12,13]. This dichotomous nature suggests that the overall output of this cluster is not universally pro- or anti-tumorigenic but is instead determined by the specific cellular environment and co-regulatory partners within the tumor. The network likely exerts its influence by fine-tuning cell fate decisions between apoptosis and cellular plasticity, which makes it a critical, though complex, determinant of cancer progression.

**Cluster-3** is organized around the *SKA3* oncogene, which interacts with other genes that are primarily involved in cell division and survival signaling. The cluster's functional profile is defined by its enrichment in processes such as the PI3K-Akt pathway and cell cycle regulation, suggesting a primary role in driving tumor proliferation and genomic instability [14]. This oncogenic drive is predominantly mediated by the hub gene *SKA3* itself, which is a critical regulator of chromosome separation and mitotic fidelity. Its overexpression disrupts normal cell division, promoting the genomic instability that is a hallmark of aggressive cancers. This function directly contributes to uncontrolled proliferation and metastasis across a spectrum of malignancies, including lung adenocarcinoma and prostate, cervical, and breast cancers [15]. The clinical significance of this activity is profound, as high levels of *SKA3* expression are a consistent marker of poor prognosis, also extending to cancers of the bladder and kidney [16,17]. Thus, the *SKA3*-centric network operates as a potent engine of tumor progression by compromising chromosomal integrity and accelerating cell division, ultimately leading to treatment-resistant and metastatic disease.

**Cluster-4** is centrally organized around the hub gene *CRYAB* (αB-crystallin), a multifunctional small heat shock protein that coordinates a network promoting robust cellular survival and immune modulation. Although the functional profile of this cluster remains largely uncharacterized in cancer but *CRYAB* plays a critical role in regulating the key apoptotic and signaling pathways, including Raf/MEK/ERK and PI3K/Akt, allowing it to inhibit pro-apoptotic proteins such as Bax, Bcl-xS, caspase-3, and p53, thereby protecting cells from stress-induced death and therapeutic challenges [18]. Beyond these cell-intrinsic survival mechanisms, *CRYAB* significantly influences the Tumor Microenvironment (TME) by modulating immune cell infiltration, positioning it as a promising biomarker for predicting immune response and prognosis across various malignancies [19]. The collective function of *CRYAB* and its associated network suggests a dual strategy whereby tumors intrinsically enhance their resilience to stress and therapy while extrinsically shaping an immunosuppressive microenvironment, ultimately facilitating immune evasion and disease progression.

**Cluster-5** is driven by the *HJURP* oncogene, a histone chaperone essential for accurate chromosome segregation during cell division [20]. Its significant upregulation across numerous cancers (e.g., liver, prostate, breast, renal, ovarian, pancreatic) is strongly linked to poor prognosis of patients [21]. *HJURP* promotes tumor proliferation, invasion, metastasis, and chemoresistance [22,23]. The collective function of *HJURP* and its associated genes is in maintaining genomic instability, which is a key mechanism enabling uncontrolled growth, metastasis, and resistance to therapy.

**Cluster-6** is centered around the transcription factor OTX1, which acts as its hub gene, and is an oncogenic factor overexpressed in various cancers, including breast, colorectal, ovarian, and laryngeal squamous carcinomas [24–26]. While the overall functional profile of this cluster in cancer remains poorly defined, *OTX1* is known to drive epithelial-mesenchymal transition (EMT), a key program underlying cancer aggressiveness. By inducing EMT, *OTX1(overexpression)* enhances cancer cell proliferation, invasion, and metastasis. Together with its associated genes, *OTX1* appears to work in a coordinated mechanism by which tumors exploit this developmental transcription factor to reprogram cells, facilitating detachment from the primary site, invasion into surrounding tissues, and spread to distant organs.

**Cluster-7** is centered on the hub gene *IQGAP3*, a Rho family GTPase that regulates key cancer processes, including proliferation, apoptosis, migration, invasion, and angiogenesis [27]. This cluster modulates multiple signaling pathways, such as MAPK, cAMP, Ras, VEGF, Wnt, and chemokine signaling etc. Overexpression of *IQGAP3* is strongly associated with poor patient prognosis [28], underscoring its critical role in driving aggressive tumour. Overall, this cluster with *IQGAP3* as a central molecular hub that integrates diverse pathways to promote tumor growth, metastasis, and angiogenesis, highlighting its significance in malignant progression.

**Cluster-8** is characterized by *RHPN1* (rhophilin Rho GTPase binding protein 1) as its hub gene. It is involved in Rho GTPase-mediated signal transduction, cytoskeletal organization, and cancer metastasis [29]. Its closely related gene, RHPN1-AS1, which is a long non-coding RNA (lncRNA) transcribed antisense to RHPN1 and functions as a potent oncogene, promoting cell proliferation, migration, and invasion in cancers such as lung adenocarcinoma, glioblastoma, ovarian, and gastric cancers [30]. Mechanistically, RHPN1-AS1 drives carcinogenesis by sponging tumor-suppressive miRNAs that prevent the degradation of oncogenic proteins like *TPX2*, which enables uncontrolled cancer cell proliferation, adhesion, and migration. Overall, this cluster highlights a coordinated RNA-protein regulatory axis that leverages Rho GTPase signaling and miRNA sponging to enhance metastatic behavior and tumor aggressiveness.

**Cluster-9** consists of a compact and X-chromosome localized network of three interacting genes: the hub gene *TCEAL2*, along with *TCEAL4* and *USP11* [31]. *TCEAL2*, a nuclear phosphoprotein that regulates gene transcription, acts as a tumor suppressor in several cancers, including ovarian, clear cell renal cell carcinoma, testicular germ cell tumors, and breast cancer, and is frequently downregulated in these malignancies [32–34]. Its expression level strongly correlates with patient prognosis. The collective activity of this small, genetically linked cluster suggests a tightly coordinated tumor-suppressive module that may regulate transcriptional programs constraining cancer initiation and progression. Overall downregulation of this hub gene in the cluster could represent a key mechanism by which tumors disable an essential protective system encoded on the X-chromosome.

**Cluster-10** also consists of only 3 genes, with *SIM2* as the hub gene being interacted with *ARNT2* and *MAGED1*. *SIM2* demonstrates a complex, context-dependent role in cancer, influenced by both genetic predisposition and tumor type. Polymorphisms in *SIM2* are linked to tumor incidence in patients with Hirschsprung disease [35], while its association with *ARNT2* implicates it in tumor progression and angiogenesis; notably, high *SIM2* expression correlates with poor prognosis in endometrial carcinoma [36]. Conversely, in breast cancer, *SIM2* appears to function as a tumor suppressor by inhibiting EMT and impeding tumor invasion [37]. A recent study further highlights the noncanonical tumor-suppressive role of *SIM2*, showing that it promotes mitochondrial oxidative phosphorylation, while its knockdown shifts energy production toward glycolysis, increases ATP levels, and ultimately enhances cell proliferation [38]. The collective function of this small cluster suggests that *SIM2* serves as a critical metabolic and phenotypic switch, whose oncogenic or tumor-suppressive impact is determined by tissue context, potentially through its interplay with its partner genes, making it a unique context-dependent modulator of cancer progression.

**Cluster-11** involves *PYGM* as the hub gene, which interacts with *PYGB* and *PPP1R3B* to reprogram cancer metabolism through glycogen and insulin pathways [39]. Overexpression of *PYGM* or *PYGB* has been reported in colorectal and pancreatic cancers [40], while *PPP1R3B* serves as a prognostic marker in stomach adenocarcinoma [41]. This cluster represents a metabolic network that supports tumor growth by deregulating energy storage and utilization. Collectively, these genes coordinate the rewiring of energy metabolism, promoting glycogen breakdown and metabolic flexibility to meet the high energetic and biosynthetic demands of proliferating tumor cells in gastrointestinal malignancies.

**Inferred genes within Clusters**

In network biology, although hub genes typically attract primary attention, inferred genes (also known as bottleneck genes) play equally important roles. These genes mediate the effects of hub genes on downstream processes, modulate context-specific responses, and act as “bridges” between functional modules, enabling cross-talk between pathways.

In this study, we also identified several key inferred genes, including *UBC, ANLN, KIF14, VIRMA, ESR1, CIT, MYC, CUL3, JUN, BIRC3, PRC1, KIF23, ECT2, PARK2*, and *TP53*, which exhibit strong connectivity with hub genes within clusters (as discussed above). Pathway enrichment analysis showed that the inferred genes are involved in key biological processes across multiple clusters.

**In cluster 1**, hub genes *CAV1, MYBL2, DES, FHL1*, and *TPX2* interacted with 220 inferred genes, primarily associated with biological processes such as gene expression, cell cycle regulation, and myogenesis. **In cluster 2**, the hub gene *RXRG* and 11 inferred genes (*RARG, PPARA, THRB, NR1H3, PPARD, NCOA2, NR1H2, NR1H4, RARB, RARA,* and *PPARG*) are associated with gene expression, signal transduction, protein metabolism, nuclear receptor signaling, and SUMOylation. **In cluster 3**, six inferred genes (*SKA2, PPP2R2D, FOXD3, SKA1, PPP2R2B,* and *ECSIT*) interact with the hub gene *SKA3*, linking it indirectly to the cell cycle, AMPK, PI3K-Akt, and Rho GTPase signaling pathways. **Cluster 4** contains six inferred genes (*CRYBA1, CRYGD, CRYAA, CRYGC, FCGR2A,* and *CRYBB2*), which interact with the hub gene *CRYAB* and collectively participate in multiple cataract-related pathways. **Cluster 5**'s hub gene *HJURP* and four inferred genes *(OIP5, MIS18A, MIS18BP1,* and *CENPA)* regulate centromere function, chromosome maintenance, kinetochore assembly, and cell cycle progression. **In cluster 6**, *OTX1* and four inferred genes *(LCE2A, KRTAP5-9, KRTAP4-2* and *KRTAP10-7)* promote keratinization. **Cluster 7**’s hub gene *IQGAP3*, along with inferred genes *RAC1*/2 and *CALM2*, modulate actin cytoskeleton organization, cAMP, Ras, VEGF, B cell receptor signaling, choline metabolism, and sphingolipid signaling. **Cluster 8**’s hub gene, *RHPN1*, interacts with *CNKSR1* and *RHOA*, governs the RHO GTPase-related signaling. **Cluster 9**’s hub gene *TCEAL2* and inferred genes like *USP11* and *TCEAL4* are involved in protein folding and DNA repair. **In cluster 10**, the hub gene *SIM2* and inferred gene *ARNT2* are linked to Hirschsprung disease pathways.

Finally, in **cluster 11**, the hub gene *PYGM* and inferred genes such as *PP1R3B* and *PYGB* regulate insulin signaling, insulin resistance, glycogen and carbohydrate metabolism, glucagon signaling, and necroptosis. By integrating these findings based on cluster enrichment analysis (mainly in clusters 1–5, 7–9, and 11), we suggest that interactions between hub and inferred genes coordinate the disruption of proliferative, signaling, and metabolic pathways. This integrated mechanism highlights the therapeutic potential of targeting hub genes, as such interventions could simultaneously influence multiple interconnected pathways via their inferred gene partners.

*************************************************************

**References**

[1] Chen S, Yu B, Du GT, Huang TY, Zhang N, Fu N. KIF18B: an important role in signaling pathways and a potential resistant target in tumor development. Discov Oncol 2024;15:430. https://doi.org/10.1007/s12672-024-01330-4.

[2] Qiu M, Wang Q, Li Q, Zhu L, Li Y, Yang S, et al. KIF18B is a Prognostic Biomarker and Correlates with Immune Infiltrates in Pan-Cancer. Front Mol Biosci 2021;8:559800. https://doi.org/10.3389/fmolb.2021.559800.

[3] Shao T, Jiang X, Bao G, Li C, Guo C. Comprehensive Analysis of the Oncogenic Role of Targeting Protein for Xklp2 (TPX2) in Human Malignancies. Dis Markers 2022;2022:1–13. https://doi.org/10.1155/2022/7571066.

[4] Wu Y-P, Ke Z-B, Zheng W-C, Chen Y-H, Zhu J-M, Lin F, et al. Kinesin family member 18B regulates the proliferation and invasion of human prostate cancer cells. Cell Death Dis 2021;12:302. https://doi.org/10.1038/s41419-021-03582-2.

[5] Jin C-Y, Du L, Nuerlan A-H, Wang X-L, Yang Y-W, Guo R. High expression of RRM2 as an independent predictive factor of poor prognosis in patients with lung adenocarcinoma. Aging 2021;13:3518–35. https://doi.org/10.18632/aging.202292.

[6] Jiang X, Li Y, Zhang N, Gao Y, Han L, Li S, et al. RRM2 silencing suppresses malignant phenotype and enhances radiosensitivity via activating cGAS/STING signaling pathway in lung adenocarcinoma. Cell Biosci 2021;11:74. https://doi.org/10.1186/s13578-021-00586-5.

[7] Zuo Z, Zhou Z, Chang Y, Liu Y, Shen Y, Li Q, et al. Ribonucleotide reductase M2 (RRM2): Regulation, function and targeting strategy in human cancer. Genes Dis 2024;11:218–33. https://doi.org/10.1016/j.gendis.2022.11.022.

[8] Pan B, Wan T, Zhou Y, Huang S, Yuan L, Jiang Y, et al. The MYBL2–CCL2 axis promotes tumor progression and resistance to anti-PD-1 therapy in ovarian cancer by inducing immunosuppressive macrophages. Cancer Cell Int 2023;23:248. https://doi.org/10.1186/s12935-023-03079-2.

[9] Chen X, Lu Y, Yu H, Du K, Zhang Y, Nan Y, et al. Pan-cancer analysis indicates that MYBL2 is associated with the prognosis and immunotherapy of multiple cancers as an oncogene. Cell Cycle 2021;20:2291–308. https://doi.org/10.1080/15384101.2021.1982494.

[10] Jiang Y, Sun M. SLC7A11: the Achilles heel of tumor? Front Immunol 2024;15:1438807. https://doi.org/10.3389/fimmu.2024.1438807.

[11] Koppula P, Zhuang L, Gan B. Cystine transporter SLC7A11/xCT in cancer: ferroptosis, nutrient dependency, and cancer therapy. Protein Cell 2021;12:599–620. https://doi.org/10.1007/s13238-020-00789-5.

[12] Li P, Zhang W, Wu Q, Zhang X, Zheng Z. Retinoid X receptor γ regulates epithelial-mesenchymal transition and tumor immune infiltration in papillary thyroid cancer tumorigenesis: an experimental and in silico study. Endocr Connect 2025;14:e250015. https://doi.org/10.1530/EC-25-0015.

[13] Joseph C, Al-Izzi S, Alsaleem M, Kurozumi S, Toss MS, Arshad M, et al. Retinoid X receptor gamma (RXRG) is an independent prognostic biomarker in ER-positive invasive breast cancer. Br J Cancer 2019;121:776–85. https://doi.org/10.1038/s41416-019-0589-0.

[14] Feng D, Wang J, Xiao Y, Wu R, Li D, Tuo Z, et al. SKA3 targeted therapies in cancer precision surgery: bridging bench discoveries to clinical applications – review article. Int J Surg 2024;110:2323–37. https://doi.org/10.1097/JS9.0000000000001123.

[15] Gao W, Zhang Y, Luo H, Niu M, Zheng X, Hu W, et al. Targeting SKA3 suppresses the proliferation and chemoresistance of laryngeal squamous cell carcinoma via impairing PLK1–AKT axis-mediated glycolysis. Cell Death Dis 2020;11:919. https://doi.org/10.1038/s41419-020-03104-6.

[16] Wang C, Liu S, Zhang X, Wang Y, Guan P, Bu F, et al. SKA3 is a prognostic biomarker and associated with immune infiltration in bladder cancer. Hereditas 2022;159:20. https://doi.org/10.1186/s41065-022-00234-z.

[17] Yamada Y, Arai T, Kojima S, Sugawara S, Kato M, Okato A, et al. Anti-tumor roles of both strands of the *miR-455* duplex: their targets *SKA1* and *SKA3* are involved in the pathogenesis of renal cell carcinoma. Oncotarget 2018;9:26638–58. https://doi.org/10.18632/oncotarget.25410.

[18] Zhang J, Liu J, Wu J, Li W, Chen Z, Yang L. Progression of the role of CRYAB in signaling pathways and cancers. OncoTargets Ther 2019;12:4129–39. https://doi.org/10.2147/OTT.S201799.

[19] Cheng L, Zou X, Wang J, Zhang J, Mo Z, Huang H. The role of CRYAB in tumor prognosis and immune infiltration: A Pan-cancer analysis. Front Surg 2023;9:1117307. https://doi.org/10.3389/fsurg.2022.1117307.

[20] Li L, Yuan Q, Chu Y-M, Jiang H-Y, Zhao J-H, Su Q, et al. Advances in holliday junction recognition protein (HJURP): Structure, molecular functions, and roles in cancer. Front Cell Dev Biol 2023;11:1106638. https://doi.org/10.3389/fcell.2023.1106638.

[21] Hu Z, Huang G, Sadanandam A, Gu S, Lenburg ME, Pai M, et al. The expression level of HJURP has an independent prognostic impact and predicts the sensitivity to radiotherapy in breast cancer. Breast Cancer Res 2010;12:R18. https://doi.org/10.1186/bcr2487.

[22] Wei Y, Ouyang G-L, Yao W-X, Zhu Y-J, Li X, Huang L-X, et al. Knockdown of HJURP inhibits non-small cell lung cancer cell proliferation, migration, and invasion by repressing Wnt/β-catenin signaling. Eur Rev Med Pharmacol Sci 2019;23:3847–56. https://doi.org/10.26355/eurrev_201905_17812.

[23] Mao M, Jia Y, Chen Y, Yang J, Xu L, Zhang X, et al. HJURP regulates cell proliferation and chemo-resistance via YAP1/NDRG1 transcriptional axis in triple-negative breast cancer. Cell Death Dis 2022;13:396. https://doi.org/10.1038/s41419-022-04833-6.

[24] Yu K, Cai X-Y, Li Q, Yang Z-B, Xiong W, Shen T, et al. OTX1 promotes colorectal cancer progression through epithelial-mesenchymal transition. Biochem Biophys Res Commun 2014;444:1–5. https://doi.org/10.1016/j.bbrc.2013.12.125.

[25] Xie H, Gong D, Zhong H, Luo J, Yu L, Gao M, et al. OTX1 promotes TNBC cell proliferation and tumor growth through the ERK pathway. Genes Dis 2025:101642. https://doi.org/10.1016/j.gendis.2025.101642.

[26] Zhang Q, Li X, Ren L, Gu X, Xiao N, Li N. OTX1 silencing suppresses ovarian cancer progression through inhibiting the JAK/STAT signaling. Tissue Cell 2023;82:102082. https://doi.org/10.1016/j.tice.2023.102082.

[27] Dongol S, Zhang Q, Qiu C, Sun C, Zhang Z, Wu H, et al. IQGAP3 promotes cancer proliferation and metastasis in high‑grade serous ovarian cancer. Oncol Lett 2020;20:1179–92. https://doi.org/10.3892/ol.2020.11664.

[28] Kumar D, Hassan MdK, Pattnaik N, Mohapatra N, Dixit M. Reduced expression of IQGAP2 and higher expression of IQGAP3 correlates with poor prognosis in cancers. PLOS ONE 2017;12:e0186977. https://doi.org/10.1371/journal.pone.0186977.

[29] Ridley AJ, Hall A. The small GTP-binding protein rho regulates the assembly of focal adhesions and actin stress fibers in response to growth factors. Cell 1992;70:389–99. https://doi.org/10.1016/0092-8674(92)90163-7.

[30] Yu F, He H, Zhou Y. Roles, biological functions, and clinical significances of RHPN1-AS1 in cancer. Pathol - Res Pract 2023;248:154589. https://doi.org/10.1016/j.prp.2023.154589.

[31] Pillutla RC, Shimamoto A, Furuichi Y, Shatkin AJ. Genomic Structure and Chromosomal Localization ofTCEAL1,a Human Gene Encoding the Nuclear Phosphoprotein p21/SIIR. Genomics 1999;56:217–20. https://doi.org/10.1006/geno.1998.5705.

[32] Zhou Y, Zhang Y, Li W, Xu J, He X, Li X, et al. TCEAL2 as a Tumor Suppressor in Renal Cell Carcinoma is Associated with the Good Prognosis of Patients. Cancer Manag Res 2020;Volume 12:9589–97. https://doi.org/10.2147/CMAR.S271647.

[33] Li X, Chen J, Hu X, Huang Y, Li Z, Zhou L, et al. Comparative mRNA and microRNA Expression Profiling of Three Genitourinary Cancers Reveals Common Hallmarks and Cancer-Specific Molecular Events. PLoS ONE 2011;6:e22570. https://doi.org/10.1371/journal.pone.0022570.

[34] Sun Y, Zhao J. Transcription Elongation Factor A (SII)-Like (TCEAL) Gene Family Member-TCEAL2: A Novel Prognostic Marker in Pan-Cancer. Cancer Inform 2022;21:11769351221126285. https://doi.org/10.1177/11769351221126285.

[35] Chatterjee A, Dutta S, Mukherjee S, Mukherjee N, Dutta A, Mukherjee A, et al. Potential contribution of SIM2 and ETS2 functional polymorphisms in Down syndrome associated malignancies. BMC Med Genet 2013;14:12. https://doi.org/10.1186/1471-2350-14-12.

[36] Nie H, Chen Y. SIM2, associated with clinicopathologic features, promotes the malignant biological behaviors of endometrial carcinoma cells. BMC Cancer 2025;25:666. https://doi.org/10.1186/s12885-025-14077-0.

[37] Kwak H-I, Gustafson T, Metz RP, Laffin B, Schedin P, Porter WW. Inhibition of breast cancer growth and invasion by single-minded 2s. Carcinogenesis 2006;28:259–66. https://doi.org/10.1093/carcin/bgl122.

[38] Wall SW, Sanchez L, Tuttle KS, Pearson SJ, Soma S, Wyatt GL, et al. Noncanonical role of singleminded-2s in mitochondrial respiratory chain formation in breast cancer. Exp Mol Med 2023;55:1046–63. https://doi.org/10.1038/s12276-023-00996-0.

[39] Migocka-Patrzałek M, Elias M. Muscle Glycogen Phosphorylase and Its Functional Partners in Health and Disease. Cells 2021;10:883. https://doi.org/10.3390/cells10040883.

[40] Xu C, Zhang S, Sun B, Yu Z, Liu* H. Machine learning identifies PYGM as a macrophage polarization-linked metabolic biomarker in rectal cancer prognosis. Front Immunol 2025;16. https://doi.org/doi: 10.3389/fimmu.2025.1639303.

[41] Zhu Y-Z, Liao X-W, Yin W, Wei H-M. Protein Phosphatase 1 Regulatory Subunit 3: A Prognostic Biomarker in Stomach Adenocarcinoma. Int J Gen Med 2022;15:1131–46. https://doi.org/10.2147/IJGM.S345978.
